# Prevalence and antimicrobial resistance patterns of nontyphoidal *Salmonella* in Ghana: a systematic review and meta-analysis

**DOI:** 10.1186/s41182-025-00731-7

**Published:** 2025-07-01

**Authors:** Patience Sarkodie-Addo, Bill Clinton Aglomasa, Eric S. Donkor

**Affiliations:** Department of Medical Microbiology, University of Ghana Medical School, Accra, Ghana

**Keywords:** Nontyphoidal *Salmonella*, Salmonellosis, Antimicrobial resistance, Meta-analysis, Systematic review, Ghana

## Abstract

**Background:**

Nontyphoidal *Salmonella* (NTS) is a foodborne pathogen of major public health concern, especially in Sub-Saharan Africa, including Ghana, where it causes invasive infections. However, data on its prevalence, antimicrobial resistance (AMR) patterns, and associated serovars in Ghana are fragmented across multiple studies.

**Objective:**

This systematic review and meta-analysis aimed to consolidate data on the prevalence, phenotypic and genotypic antimicrobial resistance profiles of NTS in Ghana.

**Methods:**

Following PRISMA guidelines, a systematic search was conducted on August 8, 2024, across four databases: PubMed, ScienceDirect, Scopus, and Web of Science. A total of 31 studies were included. A random-effects model was used to estimate the pooled prevalence of NTS and the resistance levels of antibiotics reported in two or more studies. Subgroup analysis, multivariate analysis, sensitivity analysis, Egger’s test, and forest plots were performed to explore variations, assess the influence of individual studies, test for publication bias, and visualize pooled estimates.

**Results:**

The pooled prevalence of NTS was estimated at 4.69% (95% CI 2.65–8.16) with high heterogeneity observed among the studies (I^2^ = 98.6%, τ^2^ = 1.22, τ = 1.10, H = 8.55, Q = 1754.02, p value < 0). Prevalence rates fluctuated over time: 6.27% (2008–2012), 2.09% (2013–2017), and 7.02% (2018–2023), with no significant trend observed (Q = 2.63, df = 2, p value = 0.27). Antimicrobial resistance (AMR) rates were high, with resistance to trimethoprim-sulfamethoxazole (56.7%), amoxicillin/ampicillin (50.8%), tetracycline (46.7%), and ampicillin (36.2%). Cefotaxime had the lowest resistance at 18.6%. *Salmonella* Typhimurium was the most identified serovar (36.7%), followed by *S.* Enteritidis (7.9%), *S.* Rubislaw (4.9%), *S.* Dublin (3.7%), and *S.* Kentucky (3.6%). Several AMR genes including *gyrA*, *gyrB*, *qnrB2,* and *qnrB19* were identified in human and food samples.

**Conclusion:**

Despite ongoing interventions, NTS remains a significant public health challenge in Ghana, with high AMR levels. The continued rise in resistance to critical antibiotics highlights the need for a One Health approach, improved diagnostics, enhanced surveillance, and targeted public health measures to control NTS and mitigate AMR.

**Supplementary Information:**

The online version contains supplementary material available at 10.1186/s41182-025-00731-7.

## Background

*Salmonella* is a major pathogen belonging to the Enterobacteriaceae family that causes gastroenteritis, bacteraemia, and fever in humans and animals. *Salmonella* can be divided into two main species: *Salmonella enterica* and *Salmonella bongori* [1]. *Salmonella enterica* (*S. enterica*) comprises over 2600 serovars, which are classified as either typhoidal or nontyphoidal depending on their ability to cause specific diseases such as acute diarrhoea and fever in humans and animals [2–4]. Nontyphoidal *Salmonella* (NTS) can be found in a wide range of hosts, including domestic and wild animals such as cattle, pigs, sheep, poultry, rodents, and reptiles [5], and it is predominantly spread through the consumption of contaminated food and water [6]. In 2010, NTS was estimated to be responsible for 93 million cases of gastroenteritis annually, resulting in 155,000 deaths [7]. Approximately 535,000 invasive NTS illnesses and 77,500 deaths were reported by the Global Burden of Disease Study 2017, with 422,000 (78.9%) diseases and 66,500 deaths (85.9%) occurring in sub-Saharan Africa [8]. The epidemiology and outcomes of NTS infections, however, vary across different regions, as they depend on access to clean water, food safety practices, access to healthcare, and underlying risk factors [9, 10].

*S*. Enteritidis and *S*. Typhimurium are the most common NTS serovars that cause nontyphoidal salmonellosis globally [11]. Nontyphoidal *Salmonella* infections are usually associated with acute diarrhoea, fever, nausea, vomiting, and abdominal cramps, and are often self-limiting [3]. However, in immunocompromised people, young children, or elderly individuals, NTS infections can lead to bacteraemia, meningitis, and other invasive diseases, which may be fatal [1].

Unfortunately, NTS remains a serious public health challenge worldwide despite several interventions, and sub-Saharan Africa bears the brunt of the high morbidity and mortality rates associated with this bacterium [12]. This has been attributed to host risk factors such as malaria, Human Immunodeficiency Virus (HIV), anaemia, sickle cell disease (SCD), and malnourishment, which contribute to the greater burden in this region than in other parts of the world [13, 14]. In Ghana and other African countries, invasive NTS (iNTS) diseases are more prevalent and are often severe, unlike in developed countries where NTS is often milder and limited to gastrointestinal illnesses [1]. Studies have shown that NTS is the leading cause of bloodstream infections in Ghana, especially in immunocompromised adults and children under the age of 5 [15, 16].

The increase in antimicrobial resistance among NTS isolates is an emerging concern, as it significantly impacts the success of treatment and increases the risk of more severe disease outcomes [17]. Multidrug resistance (MDR), defined as non-susceptibility to three or more classes of antibiotics to which they were previously susceptible to, has also been widely reported in NTS isolates across countries in sub-Saharan Africa [14]. A recent systematic review revealed that 75% of all NTS strains isolated from this region since 2001 were multidrug resistant [18]. Disturbingly, resistance has been reported not only to antibiotics that are used as first-choice treatment options for iNTS infections, such as chloramphenicol, ampicillin, and trimethoprim/sulfamethoxazole, but also to third-generation cephalosporins and fluoroquinolones, which are classified by the World Health Organization (WHO) as critically important antibiotics for human medicine [18, 19].

Data on the prevalence and antimicrobial resistance levels of NTS in Ghana are limited and fragmented across several studies, hindering accurate estimation of the national burden. This information is essential for proper planning and the implementation of effective solutions, as healthcare experts and policy makers rely on it to make informed decisions on prevention, control, and treatment strategies [20, 21]. The aim of this systematic review and meta-analysis, therefore, was to consolidate data on the prevalence, antimicrobial resistance levels, and specific serovars associated with NTS infections in Ghana.

## Methods

### Literature search

To comprehensively search for peer-reviewed literature, four databases were used: PubMed, Scopus, Web of Science and ScienceDirect. A combination of relevant search terms, such as “*Salmonella*”, “nontyphoidal *Salmonella*”, “salmonellosis”, “prevalence”, “antibiotic susceptibility”, “antibiotic resistance”, and “Ghana” was employed. The search was conducted on 8th August 2024.

### Inclusion and exclusion criteria

The protocol for the study was registered in the Open Science Framework (OSF) repository and is accessible via: https://doi.org/10.17605/OSF.IO/J68Z2. The eligibility of the articles for this study was determined via the Preferred Reporting Items for Systematic Reviews and Meta-analyses (PRISMA) 2020 guidelines [22]. This systematic review adopted the CoCoPop (Context, Condition, Population, and Outcome) framework, which guided our search strategy, study selection, and data collection processes. Articles were initially screened based on their titles and abstracts for the following requirements: NTS prevalence, isolated serovars, antibacterial resistance levels, and the presence of antibacterial resistance genes. Articles that published data on zoonotic *Salmonella* (such as *S*. arizonae and *S*. salamae) were also included. Studies were excluded if they lacked data on the prevalence of NTS or antibacterial resistance levels. Additionally, studies were excluded if they were not written in English or were published before the year 2000. Duplicates, poster presentations, conference abstracts, review articles, and book chapters were also excluded.

### Data collection

Each article was examined, and data were extracted. The following information was retrieved from the studies: name of the authors, year of publication, year of sample collection, study subjects (humans, animals and/or their environments), location of study, type of sample (blood, faecal, dust, carcass, water), number of samples, number of NTS isolates, NTS prevalence, levels of antibacterial resistance among the isolates, and serovars detected.

### Study quality

The quality of each study was evaluated using the Joanna Briggs Institute (JBI) Critical Appraisal Tools Checklist for prevalence studies [23], with a slight modification. The checklist comprised eight questions, with each question receiving a score of 1 for a "yes" response and 0 for a "no" or "unclear" response. Studies with a score of 80–100% (“yes” responses for 7–8 questions) were classified as low risk of bias (high quality), whilst studies which satisfied 4–6 categories (50–79%) were classified as moderate risk of bias (moderate quality). Studies with a score of < 50% were considered as high risk of bias (poor quality) (Supplementary File 1-Table 1).

### Statistical analysis

Only studies that presented data on the prevalence of NTS and/or phenotypic antimicrobial resistance data were included in the meta-analyses. The meta-analysis was conducted using R software (version 4.4.2) and the meta package (version 4.20–2). A random-effects model employing the DerSimonian-Laird (DL) method was used to account for between-study heterogeneity. To ensure robust pooled estimates despite high heterogeneity, logit transformation was applied to stabilize variances. Pooled prevalence estimates of nontyphoidal *Salmonella* (NTS) and their antimicrobial resistance profiles were calculated, with 95% confidence intervals (CIs) determined using the Clopper-Pearson method. Heterogeneity among studies was assessed using τ^2^ for between-study variance, I^2^ for the proportion of heterogeneity attributable to variability between studies, and the H index as a measure of heterogeneity. Prediction intervals were computed to estimate the likely range of prevalence in similar future studies, providing a broader context for the results. Subgroup analyses were performed to explore potential sources of heterogeneity in sample types (human, animal, or environmental), year of publication, and other variables. Heterogeneity was classified as substantially high, high, moderate or low if the I^2^ statistic was respectively > 75%, 50–75%, 25–50% and 0–25%. Additionally, sensitivity analyses were conducted to evaluate the robustness of the findings by excluding individual studies and monitoring changes in heterogeneity metrics. Publication bias was assessed using Egger's test, which was conducted on pooled estimates derived from datasets that included more than 10 studies. This threshold was applied because Egger's test lacks sufficient statistical power with fewer than 10 studies, making it less reliable and increasing the likelihood of false-positive or false-negative results. A cut-off value of 0.05 or less was used to determine statistical significance.

## Results

**Fig. 1:**
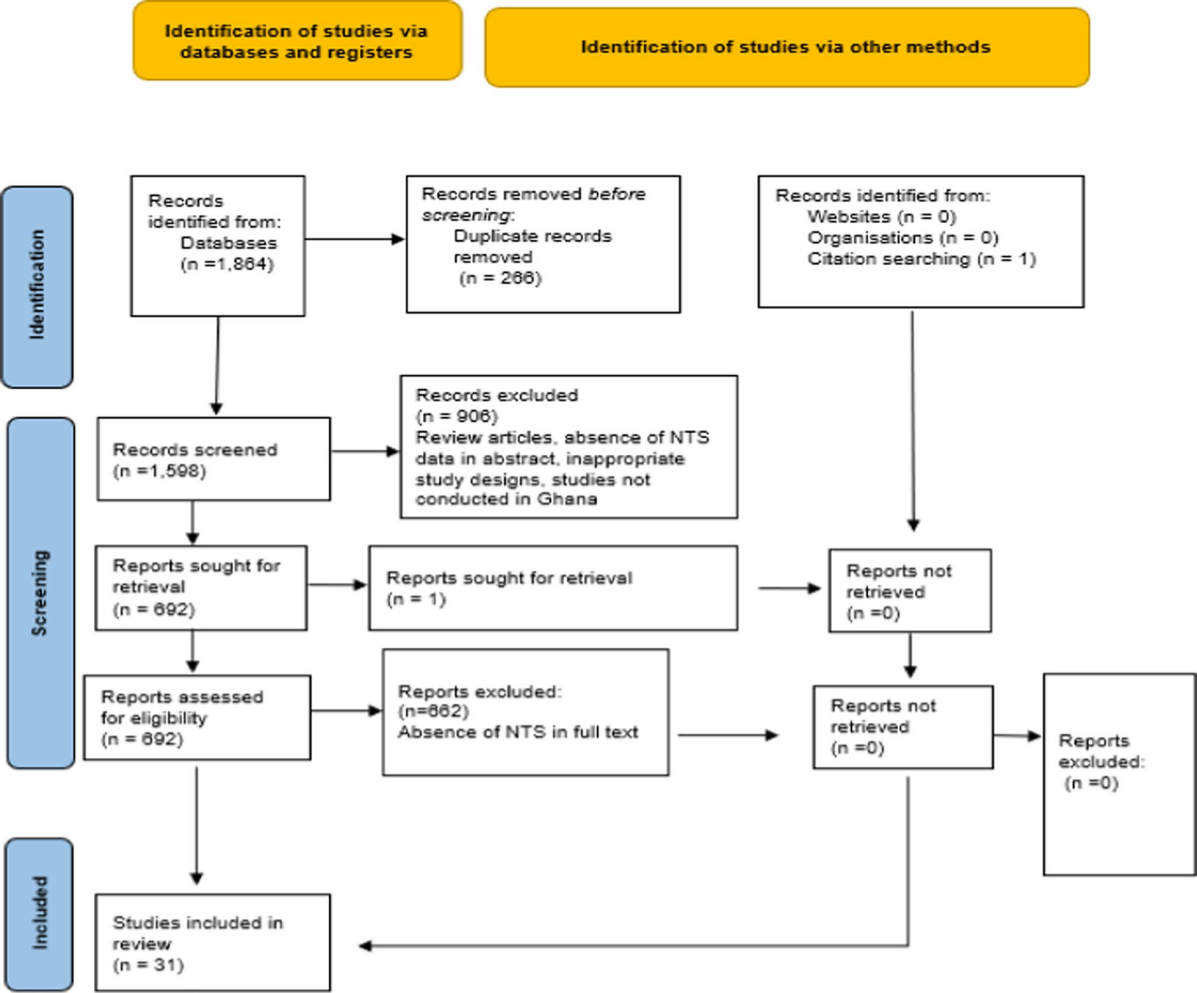
A PRISMA flow chart of the study search and study selection

A total of 1864 articles were identified from the four databases. Two hundred and sixty-six articles were removed after deduplication. Of the 1598 articles that remained, 906 were omitted because they were not research articles (e.g., conference abstracts, poster presentations, reviews or book chapters), were published in a language other than English, or did not have their full text available. Six hundred and ninety-two articles were then reviewed to determine if they met the inclusion criteria. A total of 662 articles were rejected for reasons such as the absence of NTS data in the abstract or full text, inappropriate study designs or the study not being conducted in Ghana. One article was included from another source. Thus, 31 studies were ultimately selected for this review and meta-analysis (Supplementary File 1-Tables 2 & 3) (Fig. 1).

### Study characteristics

A total of fourteen studies [16, 24–36] conducted research on NTS in human samples only, six studies investigated NTS in food samples only [37–42] and four studies investigated NTS in environmental samples only [43–46]. Two studies included NTS in both human and environmental samples [15, 48], whereas only one study investigated NTS in animal and environmental samples [47] (Supplementary File 1-Table 2).

The majority of the studies were conducted in the Middle belt of Ghana [15, 16, 24, 27–29, 31, 34–36, 40, 43–45]. Five studies were carried out in the Lower belt of the country [30, 37, 41, 42, 46], whereas three studies were conducted in the Upper belt [25, 38, 48]. Five studies were conducted in locations across all three belts of Ghana [26, 32, 33, 39, 47] (Supplementary File 1-Table 2).

Eleven studies used agglutination for serovar identification [15, 24, 25, 30, 37, 42, 43, 45–48], three studies used whole genome sequencing (WGS) [38, 39, 41], and two studies used MALDI-ToF MS [28, 40]. One study used both polymerase chain reaction (PCR) and agglutination [27]. Ten studies did not specify the method used [16, 26, 29, 31–36, 44] (Supplementary File 1- Table 2).

Twenty-two studies performed antimicrobial susceptibility testing. Sixteen articles used disk diffusion [15, 16, 24, 27, 30–34, 36, 38, 40, 45–48], and one study used only broth microdilution [39]. One study investigated antimicrobial resistance using a combination of disk diffusion and broth microdilution [25], and another used a combination of disk diffusion and a BP Phoenix M50 analyzer [26]. The remaining studies used VITEK [28, 41]; however, although one study conducted antimicrobial susceptibility testing, the authors did not state the method used [43] (Supplementary File 1-Table 2).

### Bloodstream infections in humans

Schwartz et al. reported a total of 128 NTS isolates from 1,032 samples taken from hospitalized children [34]. In a retrospective study conducted at a tertiary care hospital in the Greater Accra region to investigate *Salmonella* in bloodstream infections, NTS was detected in 115/23,708 samples [30]. NTS was detected in 24/2,306 samples of children younger than 15 years in the suburban belt of Kumasi, Ghana’s second-largest city [35]. In another notable study, the rate of NTS in rural children attending a hospital in Asante Akim Agogo (AAN) was found to be almost three times higher than that in urban children attending the same hospital [29]. Nontyphoidal *Salmonella* was reported to be the most frequently isolated Enterobacteriaceae (n = 215) causing bloodstream infections in patients of all ages in a 2007–2012 study [28]. A similar study confirmed the presence of NTS in 196/5211 blood culture samples [27]. In a surveillance study conducted in 18 laboratories across the country, NTS was detected in 0.06% of the samples [33]. Blood samples were taken from hospitalized, febrile children under the age of 15 and investigated for NTS, with a reported prevalence of 6% [15]. Among 133 biobanked blood samples taken from the Kumasi Center for Collaborative Research in Tropical Medicine, NTS was detected in 16 [24]. In another multicountry study seeking to determine the distribution and characterization of extended-spectrum β-lactamase–associated Gram-negative bacteria, 118 NTS isolates were recovered from febrile patients in Ghana [36]. A study involving four countries, including Ghana, reported NTS in 55/1,238 blood samples processed [31]. In a multicentre antimicrobial resistance (AMR) surveillance study, 18 NTS isolates were detected from 334 Gram-negative isolates submitted by various sentinel sites in the country [26].

### Gastroenteric infections in humans

In a study conducted between November 2005 and January 2006 in northern Ghana, NTS was detected in 5/367 faecal samples taken from children with or without acute diarrhoea [25]. Among 91 faecal samples obtained from both inpatients and outpatients attending a teaching hospital in a neglected area of northern Ghana, 3 NTS isolates were identified [48]. A retrospective study carried out with stool samples from two hospitals in the Ashanti region identified 9/418 samples as NTS [24]. As part of a multicentre surveillance study in Sub-Saharan Africa, 5 NTS isolates were identified from 232 stool samples collected from febrile patients in the AAN District [31].

### NTS in food

In the Nima-Kotobabi-Pig Farm-Accra New Town subarea of Accra, NTS was isolated from light soup, a common street food in the country [42]. NTS was detected in 11.9% of raw wagashi samples obtained from informal milk markets in Accra [41]. A study carried out from May to December 2015 confirmed 17 NTS isolates from 200 samples taken from local Ghanaian poultry (fresh meat) and imported frozen meat [40]. Among 44 *S*. *enterica* isolates obtained from meats in the Tamale metropolis, 16 were confirmed to be NTS [38]. Six out of 80 raw beef samples from Ashaiman Municipality were identified as *S*. Typhimurium [37]. A cross-sectional study carried out in informal markets in Accra, Kumasi and Tamale detected 21 NTS isolates from 384 egg and eggshell samples [39].

### NTS in animals and their environment

Nontyphoidal *Salmonella* was detected in 94/200 samples taken from egg-laying hens, broilers, and their environment. The samples collected included feed, faecal sock, drinking water, dust, and skin neck [47].

### NTS in the environment

In environmental samples, *S*. choleraesuis ssp. Arizonae was isolated from 2% of water samples taken from the Barekese Reservoir [44]. Drinking water from dug wells in Asante Akyem District was investigated for *Salmonella*, and NTS was detected at a prevalence of 6.5% [15]. A study on *Salmonella* in chicken carcasses and their environment reported a prevalence of 34.4%. The most isolated serovars were *S*. Typhimurium, *S.* Infantis, *S.* Enteritidis, and *S.* Newport [46]. The prevalence of NTS isolated from farm environments in two towns in the Ashanti Region was reported to be 6% [45].

### Distribution of serovars

**Fig. 2:**
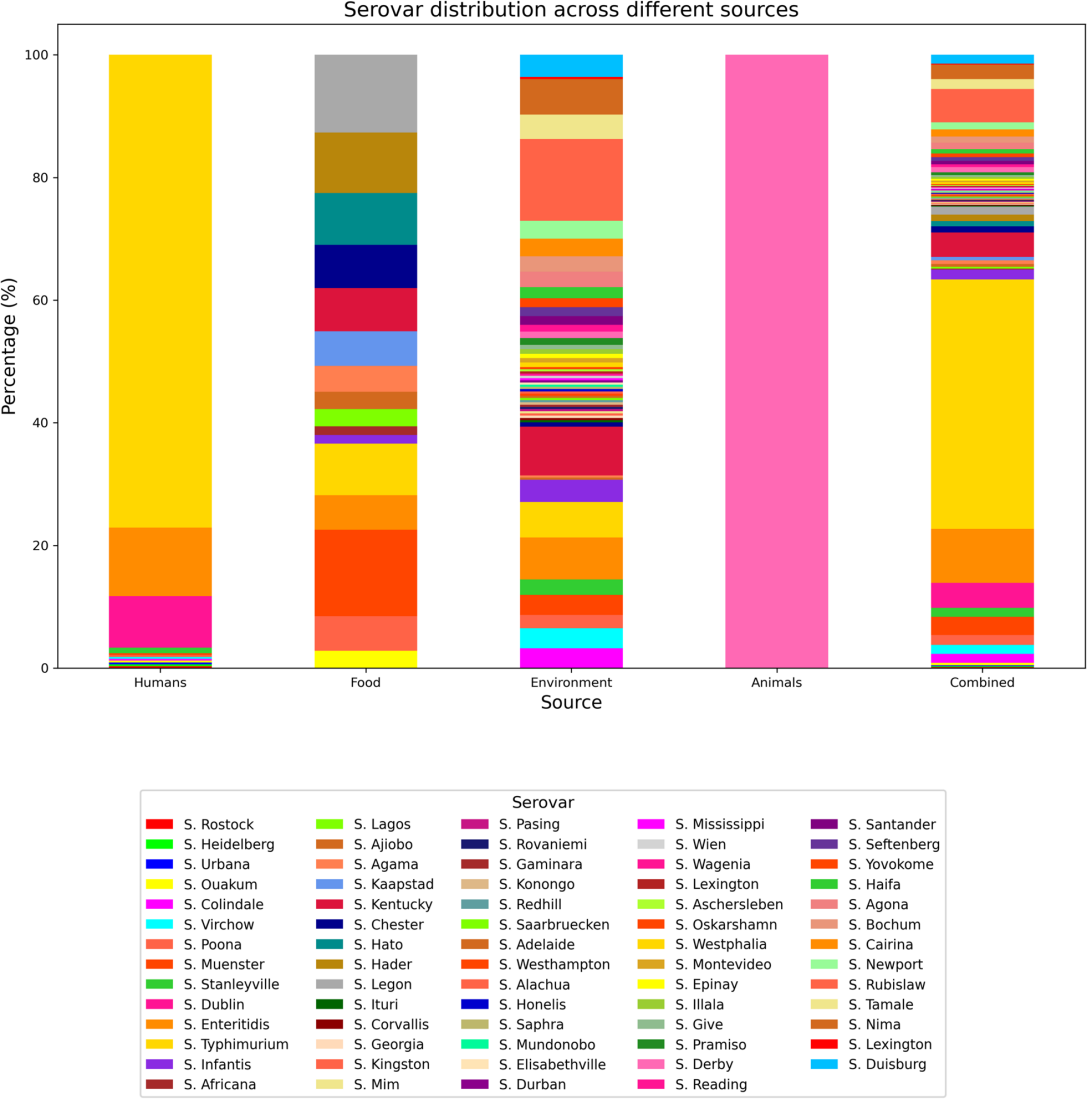
Serovar distribution across different categories

Sixteen studies included data on the type of serovars identified [15, 24, 25, 27, 37–48]. In this review, a total of 757 isolates were tested to identify their serotypes, and sixty-nine serovars were confirmed (Fig. 2). *S*. Typhimurium was the most commonly identified serovar (36.7%, n = 278), followed by *S*. Enteritidis (7.9%, n = 60), S. Rubislaw (4.9%, n = 37), *S*. Dublin (3.7%, n = 28), *S*. Kentucky (3.6%, n = 27), *S.* Muenster (2.6%, n = 20), *S.* Nima (2.1%, n = 16), *S*. Tamale (1.5%, n = 11), *S*. Infantis (1.5%, n = 11), *S*. Virchow (1.5%, n = 11), and *S*. Poona (1.5%, n = 11). Fifty-seven isolates (7.5%) were either untypable or lost during processing. A total of 69 serovars were identified in the systematic review, revealing variations in serovar distribution across sources. The greatest variety of serovars was recorded from the environment, with 60 out of the 69 identified serovars represented. Sixteen serovars were identified from food samples, while twelve serovars were detected from human samples. Only one serovar was detected from animal samples. (Fig. 2).

Humans contributed 332 *non-typhoidal Salmonella (NTS)* isolates from 12 unique serovars. Food contributed 71 isolates from 16 unique serovars. The environment contributed 277 NTS isolates from 60 unique serovars, while only one unique serovar was identified from 3 NTS isolates from animal sources.

### Pooled prevalence of NTS in Ghana

**Fig. 3:**
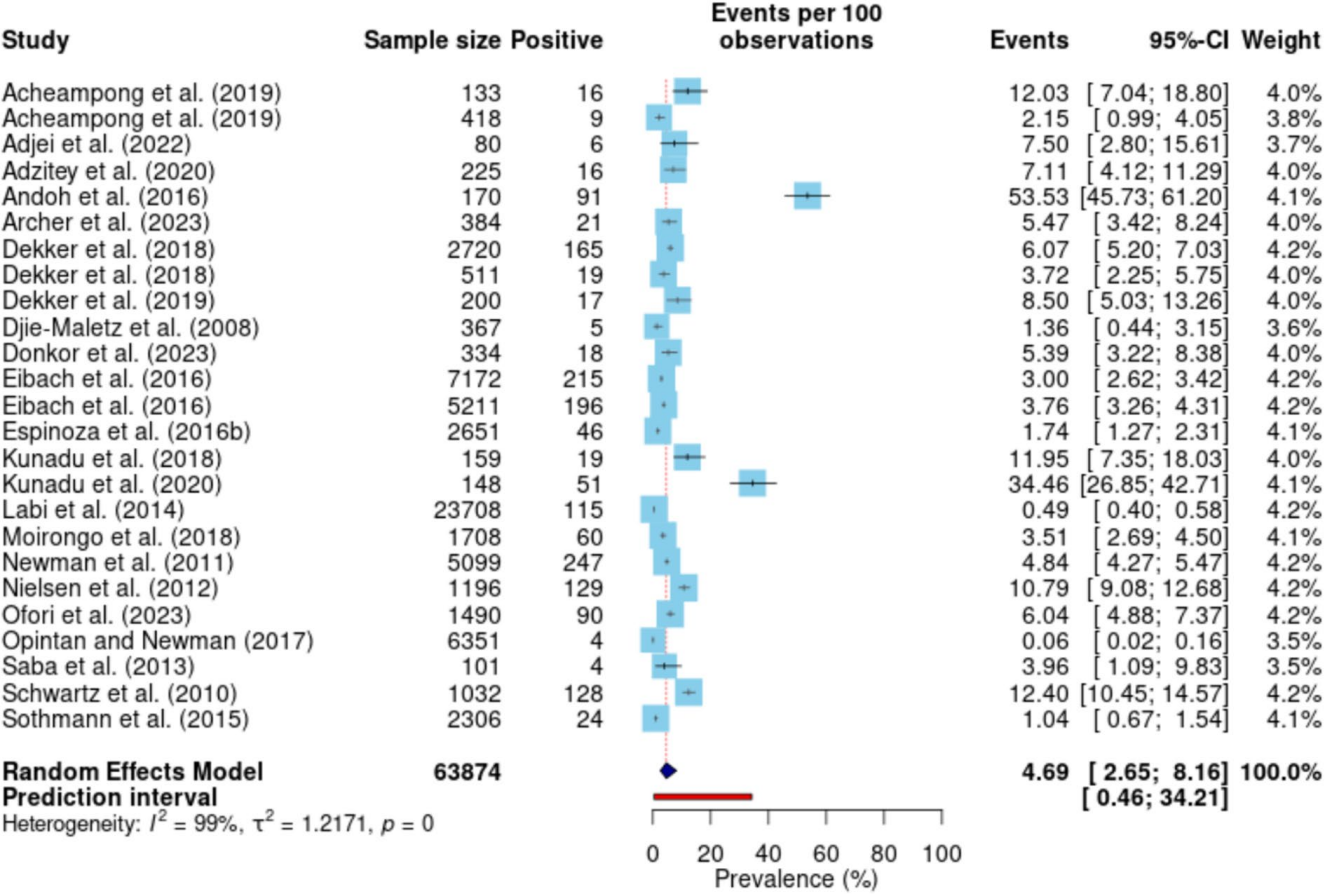
Pooled prevalence of NTS in Ghana

A total of 21 studies (with 25 data points) were included in this meta-analysis, comprising 63,874 observations and 1711 events. The pooled prevalence of NTS was estimated at 4.69% (95% CI 2.65–8.16) (Fig. 3). The prediction interval, which accounts for expected range of prevalence in similar future studies, ranged from 0.46% to 34.21%, underscoring variability in prevalence estimates across studies. The heterogeneity of the included studies was substantial, with a calculated I^2^ value of 98.6% (95% CI 98.4–98.8) and a corresponding H value of 8.55 (95% CI 7.89–9.26). The between-study variance (τ^2^) was estimated at 1.22, and the overall heterogeneity test was significant (Q = 1754.02, p < 0.001).

The distribution of prevalence rates across studies revealed several high-prevalence estimates, particularly from studies such as Andoh et al. (2016) reporting 53.53% (95% CI 45.73 –61.20) and Kunadu et al. (2020) reporting 34.46% (95% CI 26.85–42.71). Conversely, studies such as Opintan and Newman (2017) and Labi et al. (2014) reported significantly lower prevalence rates of 0.06% (95% CI 0.01–0.16) and 0.48% (95% CI 0.40–0.58), respectively.

### Influential analysis on prevalence of NTS

Influential analysis showed that omitting individual studies did not significantly alter the pooled prevalence estimate, which remained within the range of 4.15% to 5.45% (SF1).

### Publication bias analysis on prevalence of NTS

To understand whether there was publication bias, a funnel plot was generated and it was symmetrical, indicating no publication bias (SF2). This finding was further confirmed with Egger’s test which showed no significance (t = 0.31, df = 23, p-value = 0.76, bias estimate = 1.05, standard error = 3.35).

### Subgroup analysis on prevalence of NTS

**Fig. 4:**
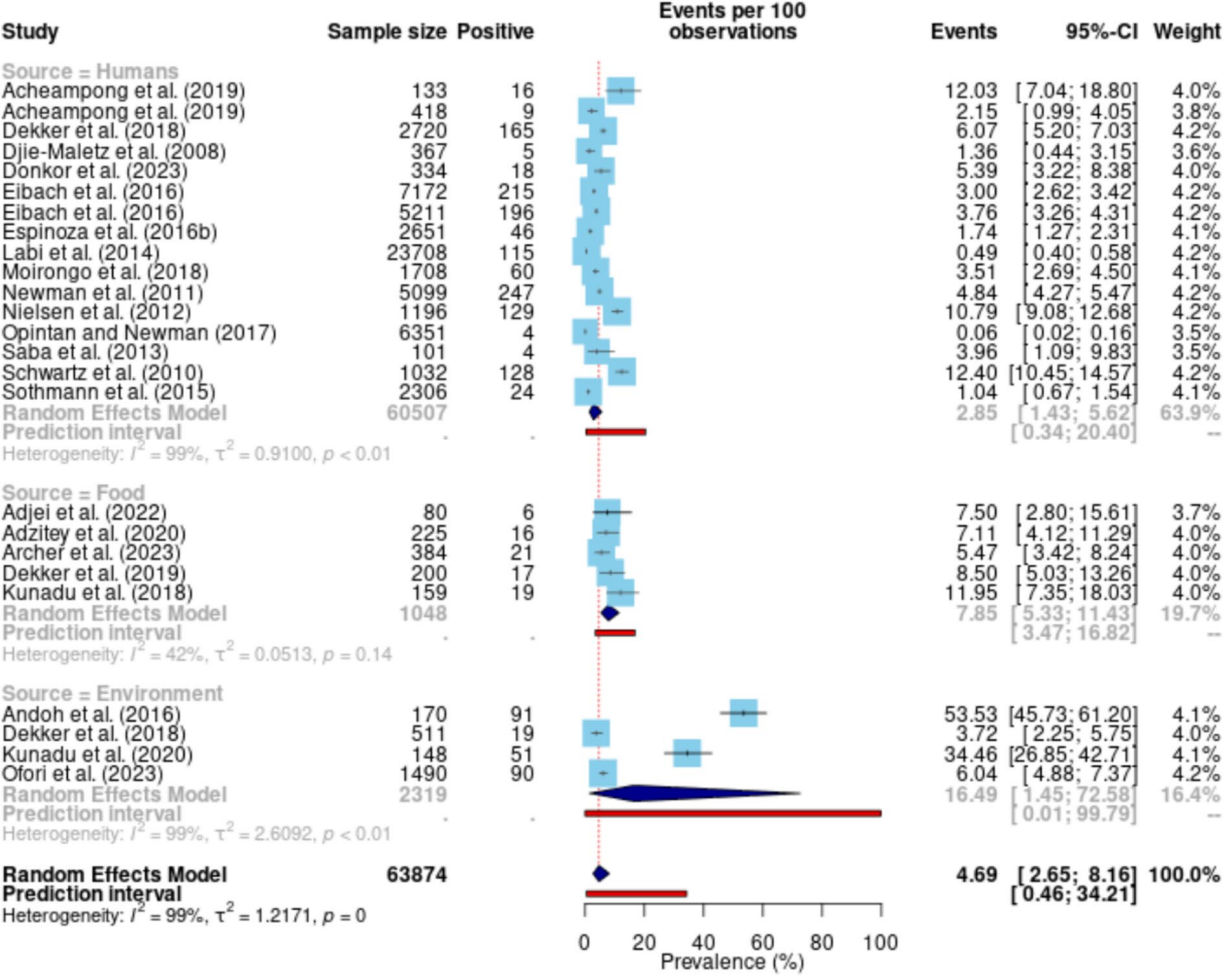
Subgroup analysis based on sources of NTS

To identify sources of heterogeneity (source of sampling, year group, study region, sample type or belts), subgroup analysis was conducted. The subgroup analysis revealed substantial variability across categories. On sources of NTS, studies involving humans had a pooled prevalence of 2.85 (95% CI 1.43–5.62; I^2^ = 98.6%; τ^2^ = 0.91, τ = 0.95, Q = 1063.92; p < 0.01) with a high heterogeneity (Fig. 4). In food-related studies, the pooled proportion was higher at 7.85 (95% CI 5.33–11.43) with moderate heterogeneity (I^2^ = 42.0%, τ^2^ = 0.05, τ = 0.23, Q = 6.90, p = 0.14). For environmental samples, the pooled proportion was the highest at 16.49 (95% CI 1.45–72.58), accompanied by very high heterogeneity (I^2^ = 99.1%, τ^2^ = 2.61, τ = 1.62, Q = 317.10, p < 0.001). A test for subgroup differences for sources indicated significant variation between groups (Q = 10.09, df = 2, p = 0.006), underscoring the distinct distribution patterns across human, food, animal, and environmental sources. Prediction intervals highlighted the range of effects expected in future studies. For human studies, the 95% prediction interval ranged from 0.34 to 20.40, whereas for food studies, the interval was narrower (3.47–16.82). For environmental studies, the prediction interval was wide, ranging from 0.01 to 99.79, reflecting high uncertainty.

For the study year groups, the pooled prevalence rates for studies conducted between 2018–2023, 2013–2017 and 2008–2012 were 7.02% (95% CI 4.50–10.80, Q = 196.94, I^2^ = 93.9%, τ^2^ = 0.49, τ = 0.70), 2.09% (95% CI 0.36–11.15, Q = 1047.05, I^2^ = 99.3%, τ^2^ = 2.18, τ = 1.48) and 6.27 (95% CI 1.38–24.21, Q = 119.39, I^2^ = 97.5%, τ^2^ = 0.40, τ = 0.63,) respectively (SF3). The test for subgroup differences revealed no significant variation in prevalence across the year groups (Q = 2.63, df = 2, p = 0.27) although prediction intervals further highlighted variability. The pooled prevalence rates for blood samples, stool samples and mixed samples were 2.78% (95% CI 0.97–7.69, Q = 1041.11, I^2^ = 99.0%, τ^2^ = 1.21, τ = 1.10,), 2.18% (95% CI 0.65–7.02, Q = 2.58, I^2^ = 22.6%, τ^2^ = 0.05, τ = 0.23) and 9.59% (95% CI 4.00–21.24, Q = 523.69, I^2^ = 98.5%, τ^2^ = 1.32, τ = 1.15), respectively (SF4). There was significant difference in prevalence across the sample types (Q = 19.72, df = 4, p = 0.0006), giving credence to the variability in the subgroups.

**Fig. 5:**
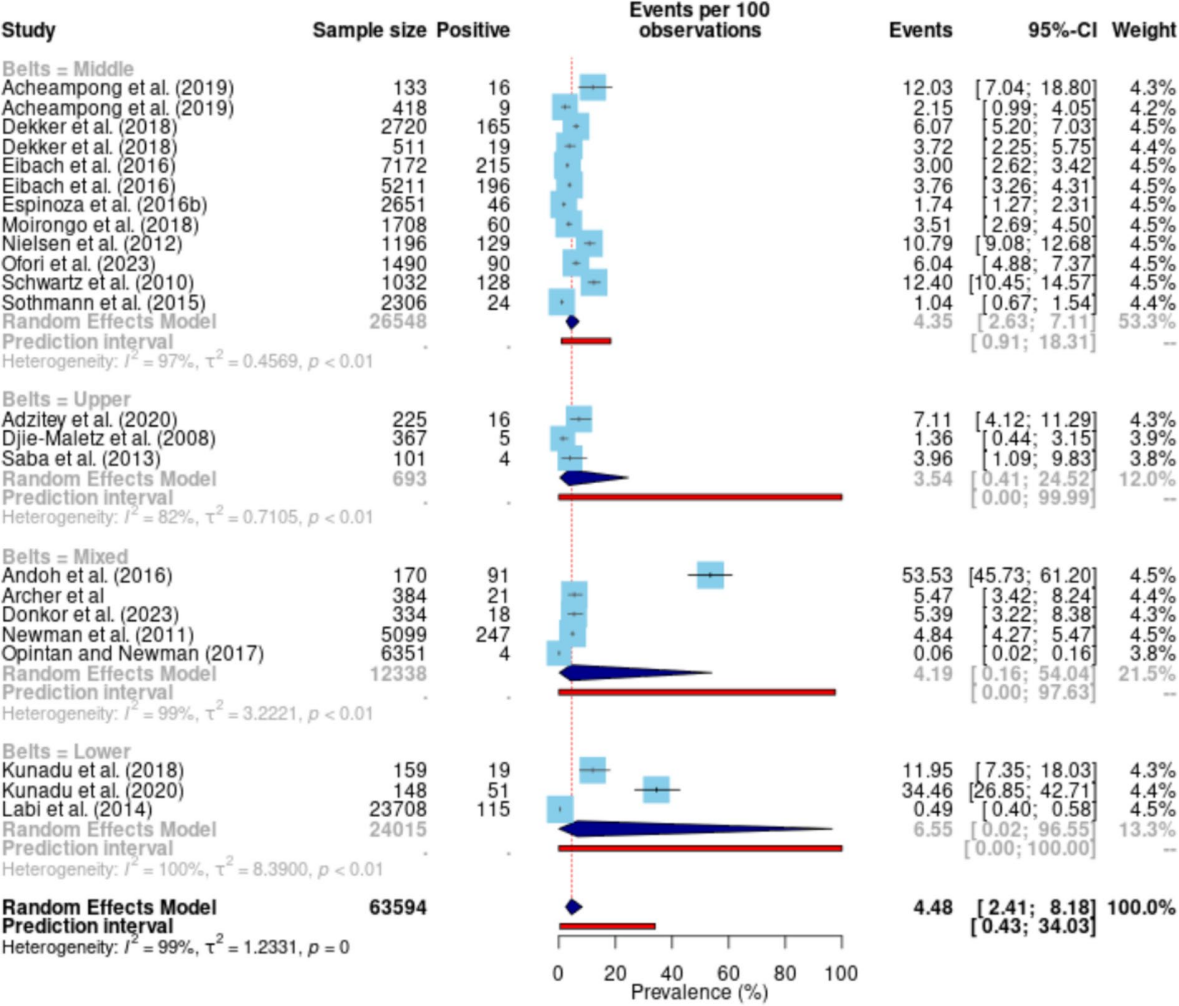
Subgroup analysis based on the locations of the studies

For the belts in which studies were conducted in Ghana, the Middle belt subgroup displayed an I^2^ of 97.2%, with an estimated prevalence of 4.57% (95% CI 2.85–7.24, Q = 424.64, τ^2^ = 0.45, τ = 0.67). Similarly, the Upper belt subgroup exhibited an I^2^ of 81.8%, an estimated prevalence of 3.53% (95% CI 0.41–24.52, Q = 10.98, τ^2^ = 0.71, τ = 0.84). The Mixed belt subgroup showed extreme heterogeneity (I^2^ = 99.1%), with a prevalence of 4.19% (95% CI 0.16–54.04, Q = 444.72, τ^2^ = 3.22, τ = 1.80). The Lower belt subgroup displayed the highest heterogeneity (I^2^ = 99.5%), with a prevalence of 6.77% (95% CI 0.31–62.71, Q = 656.61, τ^2^ = 7.71, τ = 2.78). The test for subgroup differences indicated no statistically significant variation in prevalence among the four subgroups (Q = 0.44, df = 3, p = 0.93). This suggests that the variable "Belts" does not substantially explain the heterogeneity observed across the studies (Fig. 5).

### Mixed effect analysis on prevalence of NTS

To investigate the factors contributing to heterogeneity, mixed-effects analyses incorporating different moderators were conducted. Across all the models, residual heterogeneity remained high. The amount of heterogeneity explained (R^2^) varied depending on the moderators included with source (15.59%, τ^2^ = 1.03, τ = 1.01, I^2^ = 98.41%, H^2^ = 63.09%) and year group (4.26%, τ^2^ = 1.17, τ = 1.08, I^2^ = 98.39%, H^2^ = 61.97%) accounting for some of the heterogeneity. The human category (estimate = − 1.9145, 95% CI − 3.36 to − 0.47, se = 0.70, p = 0.01) was statistically significant and showed a negative association relative to the reference category (environment), suggesting that studies involving human samples had a lower effect estimate than those in the environment. Another model, combining year group and source as moderators, increased the heterogeneity explained to 22.07% (τ^2^ = 0.95, τ = 0.97, I^2^ = 98.04%, H^2^ = 50.90%). However, the test for residual heterogeneity remained highly significant (QE(df = 20) = 1017.9061, p < 0.0001), confirming substantial variability among studies. The overall test for moderators (year group and source) was significant (F(df1 = 4, df2 = 20) = 3.0602, p = 0.04). These results highlight that while the inclusion of moderators improved the model's explanatory power to some extent, considerable unexplained heterogeneity persists. All results used in arriving at this conclusion for the meta-analysis on prevalence can be found in Additional file 1.

### Phenotypic resistance

For antibiotics used singly across studies in this review, 74.3% of NTS isolates obtained from human blood samples were reported to be resistant to amoxyclav [34]. Only 1 out of 71 NTS isolates (1.4%) was reported to be resistant to amikacin [30]. Similarly, 1 out of 26 NTS isolates (3.8%) was resistant to ampicillin/sulbactam, as reported by Dekker et al. [43]. The resistance rates of NTS from environmental samples to doripenem, erythromycin, oxacillin, pefloxacin and tigecycline were reported to be 47%, 94%, 100%, 67% and 14%, respectively [46]. Colistin resistance was detected in 2/16 (13%) NTS obtained from eggs [39]. NTS strains isolated from human blood samples presented resistance rates of 16.7%, 23.5%, 27.8% and 16.7% to meropenem, azithromycin, ceftazidime, and ertapenem, respectively [26].

A meta-analysis was conducted to assess the phenotypic resistance of NTS to various antibiotics in Ghana. The analysis included data on chloramphenicol, trimethoprim-sulfamethoxazole, tetracycline, ciprofloxacin, cefuroxime, amoxicillin-clavulanate, amoxicillin/ampicillin, gentamicin, nalidixic acid, cefotaxime, ampicillin, sulfamethoxazole and trimethoprim. Resistance to chloramphenicol, with 649 observations and 282 events, ranged from 3.92% (95% CI 0.48–13.46) in Kunadu et al. (2020) to 82.68% (95% CI 74.96–88.81) in Nielsen et al. (2012), yielding a pooled resistance of 25.64% (95% CI 7.65–58.93, I^2^ = 95.8%, τ^2^ = 2.83, τ = 1.68, Q = 190.35; p < 0.0001, H = 4.88) (SF5). Similarly, trimethoprim-sulfamethoxazole resistance, based on seven studies with 481 observations and 284 events, was pooled at 56.74% (95% CI 35.32–75.90, I^2^ = 91.5%, τ^2^ = 0.91, τ = 0.95, Q = 70.34; p < 0.0001, H = 3.42), with significant variability in individual estimates (SF6). Tetracycline resistance from nine studies involving 407 observations and 190 events was 46.7% (95% CI 17.1–78.8, I^2^ = 91.9%; τ^2^ = 1.87, τ = 1.37, Q = 98.39; p < 0.0001, H = 3.51) (SF7).

Ciprofloxacin resistance, calculated from 762 observations and 231 events across nine studies, was pooled at 33.2% (95% CI 6.1–79.1%, I^2^ = 92.6%, τ^2^ = 2.02, τ = 1.42, Q = 108.82; p < 0.0001, H = 3.69), while cefuroxime resistance from three studies involving 182 observations and 65 events was 32.5% (95% CI 0.4–98.3%, I^2^ = 89.7%, τ^2^ = 2.67, τ = 1.64, Q = 19.49; p < 0.0001, H = 3.12) (SF8, SF9). Amoxicillin-clavulanate resistance, based on five studies with 388 observations and 150 events, was pooled at 34.3% (95% CI 10.1–70.7%, I^2^ = 95.4%, τ^2^ = 1.67, τ = 1.29, Q = 86.63; p < 0.0001, H = 4.65) (SF10). Amoxicillin/ampicillin resistance, pooled from two studies, was 50.8% (95% CI 0.0–100%, I^2^ = 94.22%, τ^2^ = 5.93, τ = 2.44, Q = 94.22; p < 0.0001, H = 9.71), demonstrating substantial heterogeneity, with individual estimates ranging from 15.4% to 85.4% (SF11). Gentamicin resistance from six studies (387 observations and 120 events) was 25.5% (95% CI 6.26–63.62%, I^2^ = 95.7%, τ^2^ = 3.28, τ = 1.81, Q = 116.80; p < 0.0001, H = 4.83) (SF12).

Resistance to nalidixic acid and cefotaxime also exhibited notable variability. Nalidixic acid resistance, based on four studies with 255 observations and 71 events, was 31.4% (95% CI 0.51–97.58%, I^2^ = 93.6%, τ^2^ = 4.63, τ = 2.15, Q = 46.58; p < 0.0001, H = 3.94), while cefotaxime resistance from four studies with 172 observations and 18 events was 18.6% (95% CI 1.06–82.89%, I^2^ = 86.9%, τ^2^ = 2.40, τ = 1.55, Q = 22.82; p < 0.0001, H = 2.76) (SF13, SF14). Ampicillin resistance, pooled from seven studies with 405 observations and 194 events, was 36.2% (95% CI 13.61–67.17%, I^2^ = 95.6%, τ^2^ = 2.42, τ = 1.56, Q = 112.40; p < 0.0001, H = 4.33) (SF15). Sulfamethoxazole resistance, pooled from two studies with 110 observations and 32 events, was 34.85% (95% CI 0.07–99.77%, I^2^ = 73.3%, τ^2^ = 0.42, τ = 0.65, Q = 3.75; p = 0.05, H = 1.94), and trimethoprim resistance, pooled from two studies with 110 observations and 26 events, was 31.37% (95% CI 0.005–99.98%, I^2^ = 84.6%, τ^2^ = 0.88, τ = 0.94, Q = 6.51; p = 0.01, H = 2.55) (SF16, SF17).

Influential analysis indicated that the majority of the pooled resistance estimates were robust, as shown in Supplementary Figs. 18 through 29. However, for amoxicillin-ampicillin, sulfamethoxazole and trimethoprim antibiotics, wider disparities were observed, likely due to the fact that each was represented by only two studies (SF18, SF27, SF29). Overall, significant variability was observed across studies for most antibiotics, underscoring the importance of surveillance systems to better understand antibiotic resistance trends in NTS in Ghana. All results from resistance meta-analysis can be found in Additional file 2.

### Genotypic resistance to antimicrobials

A novel Gyrase B (*gyrB*) mutation in *S*. Typhimurium related to fluoroquinolone resistance was detected in a malnourished 17-month-old girl suffering from a bloodstream infection [49]. Single base-pair mutations were identified in Gyrase A (*gyrA*) at codon 87 in nine out of the ten *S.* Enteritidis strains. This type of mutation was again observed in *gyrA* at codon 83 and *gyrB* at codon 466 in 3 *S.* Typhimurium strains [27]. Genes associated with ESBL production (bla_*TEM52*-B_ and bla_*CTX*-*M15*_) were reported in two *S*. Virchow and *S*. Poona strains which were resistant to cephalosporins. These resistance genes were found on IncX1 and TrfA/IncHI2/IncHI2A type plasmids, respectively [50]. The occurrence of single *gyrA* mutations at codon 87 was detected in 13 NTS isolates. Eleven isolates carried sulfonamide resistance genes (mostly *sul2*), ten isolates carried *strA* and *strB* genes which are associated with streptomycin resistance, and another nine isolates harboured tetracycline resistance genes (mostly *tetA*) [51]. In a study involving NTS detection in humans, mutations known to confer resistance to ciprofloxacin were discovered at position D87G on the *gyrA* gene [31]. The presence of the quinolone resistance gene *qnrS* was detected in 2 NTS isolates from the blood and oropharynx of patients. Five different nonsynonymous mutations were also detected during the analysis of *gyrA*. The most common mutation (Ile203Ser) was observed in 12 out of the 13 tested strains. The *gyrB* gene was found to have one nonsynonymous mutation in 3 out of the 13 strains, with this mutation occurring at codon 601. However, no mutations were detected in the *parC* and *parE* genes [24]. In another study, four *S.* Poona strains carried the *aacA4* gene, which is related to aminoglycoside resistance. Several mutations were detected in 3 *S*. Kentucky strains, with double mutations occurring in *gyrA* and *parC*. Five *S.* Poona and *S*. Kentucky strains were found to carry the plasmid mediated quinolone resistance (PMQR) genes *qnrB2* and *qnrB19* [40]. The presence of a point mutation in Quinolone-Resistance-Determining-Regions (QRDRs) of *gyrA* was associated with high nalidixic acid MIC values in *S*. Enteritidis and *S*. Hadar. *S*. Chester isolate G10 was discovered to carry the *qnrB19* gene. In *S*. Chester*,* tetracycline, trimethoprim and sulfamethoxazole resistance were associated with *tetA*, *dfrA14* and *sul2* genes, respectively. Furthermore, aminoglycoside resistance genes (*aph (3’’)-Ib* and *aph(6)-Id)* were also detected in *S*. Chester. In *S*. Enteritidis, genes responsible for trimethoprim resistance were *dfrA1*, *sul1* and *sul2*, whilst chloramphenicol resistance was associated with *catA1*. The *aaaA1* gene responsible for aminoglycoside resistance was also discovered in *S*. Enteritidis. The fosfomycin resistance gene *fosA7*.*2* was found in *S*. Ajiobo and *S*. I 4:b:-. All the NTS isolates carried *aac6*-*Iy*, a chromosomal-encoded aminoglycoside acetyltransferase [39]. A study reported amino acid substitutions at positions S83Y on *gyrA* and E466D on *gyrB* in invasive *S*. Typhimurium clades [52].

### Risk of publication bias

To evaluate the potential impact of publication bias on our pooled prevalence estimate, we employed both graphical and statistical methods. First, we constructed a funnel plot to visually inspect the symmetry of the included studies’ effect sizes (SF2). The funnel plot did not reveal any obvious asymmetry, suggesting minimal publication bias. In addition, we performed Egger’s regression test, which quantitatively assesses the relationship between study size and effect estimate. The test yielded a t-value of 0.31 with 23 degrees of freedom (p = 0.76), indicating no statistically significant evidence of small-study effects. This result corroborates the visual impression obtained from the funnel plot. To further validate the robustness of our findings, we conducted an influential analysis by sequentially omitting individual studies. The pooled prevalence estimate remained stable, fluctuating only between 4.15% and 5.45% (SF1). This consistency reinforces that our overall result is not driven by any single study and that publication bias is unlikely to have influenced the findings.

For pooled estimates derived from the antibiotics datasets, because studies that reported on each antibiotic were fewer than 10 studies, publication bias was not assessed using Egger's test or funnel plots due to insufficient statistical power, which could lead to unreliable results. However, these were assessed using influential studies to understand how some studies affected the overall estimates. While this represents a limitation, we followed rigorous inclusion and exclusion criteria during the literature search and selection process to minimize the risk of bias. Furthermore, we ensured that all eligible studies were included to provide as comprehensive an analysis as possible.

## Discussion

The study screened 1,864 articles to identify relevant research on nontyphoidal *Salmonella* (NTS) in Ghana. After applying the inclusion and exclusion criteria, 31 studies were selected. The majority of studies focused on human samples [15, 16, 24–36, 48–52], which reflects the global emphasis on NTS as a human pathogen [53, 54]. However, this also highlights a research gap in food, environmental, and animal samples in Ghana. Similar gaps have also been reported in other African countries, such as Nigeria and Kenya, where research has predominantly focused on human infections rather than animal, food and environmental sources [55, 56]. The geographic distribution of the studies revealed that most were concentrated in the middle belt of Ghana [16, 24, 27–29, 31, 34–36, 40, 43–45, 57], with some representation from the lower [37, 41, 42, 46] and upper belts [25, 38, 48]. This geographic concentration could be due to many factors such as the fact that NTS is endemic in that region, or there is a lack of potable water, necessitating the high burden of NTS in that region [58]. As such, there is an opportunity for future research to extend investigations into the upper and lower belts, to understand NTS prevalence.

The pooled prevalence of NTS in Ghana, from human, animal, food and environmental sources, was estimated at 4.69% (95% CI 2.65–8.16%), highlighting the burden of NTS infections within the country. This finding is slightly higher than the 2–5% prevalence reported in Asia, and this is not surprising as Ghana is a country with a relatively high burden of infectious diseases [4, 59, 60]. However, the wide prediction interval (0.47%–32.78%) and substantial heterogeneity (I^2^ = 98.5%) suggest considerable variability in prevalence across studies. The high heterogeneity observed suggests that most of the variability in the pooled estimates is attributable to differences between studies rather than chance. This heterogeneity may be due to variations in study populations, methodological differences and temporal factors, such as changes in antibiotic usage and healthcare practices. For instance, differences in demographic characteristics, disease severity, geographical settings, study design, sample size, diagnostic methods, and antibiotic susceptibility testing protocols likely contributed to the observed variability [61].

High-prevalence estimates, such as those reported by Andoh et al. [47] (53.53%) and Kunadu et al. [46] (34.46%), were markedly distinct from studies such as Opintan and Newman [33] and Labi et al. [30], which reported much lower rates of 0.06% and 0.48%, respectively. These discrepancies may be attributed to the source of samples and year group, as explained by the mixed effects model or other differences including regional hotspots of infection, and temporal factors influencing disease dynamics. Furthermore, influential analyses demonstrated that individual studies did not disproportionately skew the pooled prevalence, indicating robustness in the overall estimate.

Subgroup analyses revealed significant variations in prevalence based on the sources of NTS. Studies involving environmental samples reported the highest pooled prevalence (16.49%, 95% CI 0.01–99.79%), compared with food (7.85%, 95% CI 5.33–11.43%), and human samples (2.85%, 95% CI 1.43–5.62%). These findings highlight the critical role of environmental reservoirs in the transmission of NTS [62], particularly in resource-limited settings where environmental contamination and inadequate sanitation may exacerbate disease spread. Again, the ability of *Salmonella* to persist in soil and water may also attribute to this key finding [63, 64]. Studies conducted in the Americas and South Asia have also reported high NTS prevalence in environmental samples [65, 66]. The significant differences in prevalence between sample types (p = 0.006) further suggest that the dynamics of NTS infections are also influenced by sample origin, with mixed samples exhibiting higher prevalence rates (9.59%, 95% CI 4.00–21.24%) than blood or stool samples. Temporal trends in the prevalence of NTS were also examined, with studies conducted between 2008–2012, 2013–2017, and 2018–2023 showing pooled prevalence estimates of 6.27 (95% CI 1.38–24.21%), 2.09 (95% CI 0.36–11.15%), and 7.02 (95% CI 4.50–10.80%), respectively. Although no significant differences were observed across the year groups (p = 0.27), the consistent presence of NTS across decades emphasizes the need for sustained surveillance and control measures [67].

Mixed-effects models incorporating moderators such as source category and year group accounted for a modest proportion of the observed heterogeneity (up to 21.30% explained variance), with environmental samples showing the strongest association with higher prevalence rates. Despite these findings, residual heterogeneity remained high, highlighting the multifactorial nature of NTS prevalence. The lack of significant results in the overall test for moderators (p = 0.67) suggests that additional unmeasured factors such as socioeconomic status, local healthcare infrastructure or regional antibiotic usage patterns may contribute to the variability.

In terms of human infections, the prevalence of NTS in bloodstream infections varied significantly across studies. For example, one study conducted in hospitals [34] reported the highest prevalence of 12.40 (n = 128/11,032), suggesting that NTS may be an underappreciated cause of bloodstream infections in Ghana. In contrast, a study by Opintan and Newman [33] detected NTS in only 0.06% of samples, indicating discrepancies that could be due to differences in the population sampled, laboratory methods, or study settings. With respect to gastroenteric infections, NTS detection in stool samples was low, potentially reflecting underreporting or misdiagnosis, especially in areas with limited diagnostic capabilities. The finding that rural children had a higher rate of NTS infections compared to urban children suggests that environmental and socioeconomic factors play a significant role in the prevalence of NTS in different populations [68], highlighting the need for better surveillance in rural areas and public health interventions to address these disparities.

The review also found that NTS was prevalent in food, particularly street food, and persisted in the environment, including poultry, meat and water sources. This points to the potential for zoonotic transmission of NTS in Ghana. The detection of NTS in meat and poultry products, as noted in studies by Adjei et al. [37] and Adzitey et al. [38], aligns with global findings that foodborne transmission is a significant pathway for NTS infections [69, 70]. These findings emphasize the importance of improving food safety practices and public health interventions aimed at reducing contamination with pathogenic bacteria [71]. Additionally, the identification of *S.* Typhimurium as the most common serovar (36.7%) among the 757 isolates analysed is consistent with global epidemiological trends although in Vietnam, *S*. Weltevreden is dominant [4, 72, 73]. This serovar, *S.* Typhimurium*,* is frequently associated with both human and animal infections [74]. The diversity of serovars observed across different environmental, food, and clinical samples underscores the ecological complexity of NTS and suggests that multiple reservoirs contribute to its persistence in Ghana, informing targeted interventions, such as focusing on *S.* Typhimurium for surveillance and control efforts.

Globally, NTS is a major cause of bloodstream and gastroenteric infections, and the findings from Ghana are in line with this [75]. However, research also highlights significant concerns regarding antimicrobial resistance (AMR) in NTS strains. Many studies, such as Donkor et al. [26], have reported a concerning rise in antimicrobial-resistant NTS strains, particularly those resistant to extended-spectrum β-lactams. The increasing prevalence of antimicrobial resistance (AMR) among NTS strains poses a major challenge for effective treatment. Resistance to chloramphenicol, for instance, ranged from 3.92% to 82.68%, with a pooled resistance estimate of 25.64% (95% CI 7.65–58.93, I^2^ = 95.8%). Similarly, resistance to trimethoprim-sulfamethoxazole and tetracycline was notably high, with pooled rates of 56.74% (95% CI 35.32–75.90, I^2^ = 91.5%) and 46.7% (95% CI 17.1–78.8, I^2^ = 91.9%), respectively. Alarmingly, resistance to ciprofloxacin, a first-line treatment for NTS infections, was also significant at 33.2% (95% CI 6.1–79.1%, I^2^ = 92.6%). These findings reflect both widespread resistance and substantial heterogeneity across studies. The high rates of resistance to critical antibiotics such as cefuroxime (32.5%; 95% CI 0.4–98.3%, I^2^ = 89.7%) and cefotaxime (18.6%; 95% CI 1.06–82.89%, I^2^ = 86.9%) further emphasize the growing threat of extended-spectrum β-lactamase-producing NTS strains in Ghana. Similarly, resistance to amoxicillin/ampicillin (50.8%; 95% CI 0.0–100%, I^2^ = 94.22%) and gentamicin (25.5%; 95% CI 6.26–63.62%, I^2^ = 95.7%) highlights the diminishing efficacy of widely used antibiotics.

Overall, the meta-analysis revealed significant variability in phenotypic resistance across studies, indicating that antibiotic resistance in NTS is a complex and a multifactorial issue in Ghana. The observed heterogeneity also underscores the importance of considering local factors, such as regional differences in antibiotic use, healthcare infrastructure, and environmental influences, when interpreting resistance patterns [76, 77]. These findings emphasize the urgent need for effective antimicrobial stewardship programs, targeted surveillance and public health interventions to mitigate the spread of antibiotic-resistant NTS in Ghana [78, 79]. Some studies used traditional methods such as agglutination, while others employed more advanced techniques such as whole genome sequencing, indicating how diagnostic approaches across both research and clinical settings have evolved over time. Enhanced surveillance efforts will improve our understanding of the true burden of NTS infections in Ghana. In terms of public health, the significant role of foodborne transmission in NTS infections, particularly through contaminated poultry, meat, and street food, calls for improved public health interventions. Policy initiatives focused on food safety, hygiene, and public health education could significantly reduce the incidence of foodborne NTS infections. The environmental contamination of water sources also highlights the need for better sanitation and water treatment practices.

Future research should focus on the role of different ecological niches, including human, animal, food, and environment, in the transmission and persistence of NTS. Longitudinal studies tracking the prevalence and evolution of NTS in multiple reservoirs could provide insight into the dynamics of transmission. Additionally, there is a need for more research on the antimicrobial resistance profiles of NTS strains in Ghana, which is critical for informing national treatment guidelines.

## Limitations

While this review provides a comprehensive understanding of the prevalence and transmission of NTS in Ghana, there are some limitations to consider. The majority of studies were conducted in the middle belt of Ghana, which could skew the results and limit the generalizability of the findings. There is also a lack of data on antimicrobial resistance in many studies, which is a critical area for further investigation.

For the pooled prevalence estimates, publication bias was thoroughly evaluated using established methods (e.g., funnel plots and Egger’s test). However, for the pooled estimates of antibiotic resistance, publication bias assessments were not conducted. This decision was based on the limited number of studies available for each antibiotic (fewer than 10), as tests for publication bias in such cases lack sufficient power and reliability. To mitigate concerns regarding the influence of individual studies on the resistance estimates, an influential analysis was performed on antibiotics having more than two estimates. This analysis examined the impact of each study on the overall pooled resistance estimates. Readers should interpret the pooled resistance estimates with caution, acknowledging that the potential for publication bias remains, given the limited dataset. Future research with a larger number of studies is needed to enable a more robust assessment of publication bias in antibiotic resistance data.

## Conclusion

This review highlights the significant burden of NTS infections in Ghana, particularly in human, food, and environmental samples. The findings underscore the need for improved diagnostic practices, better surveillance systems, and targeted public health interventions to reduce the burden of NTS. One Health policies must also be implemented and adhered to, in order to address the challenges faced from this harmful pathogen. This review provides a foundation for further research into the transmission dynamics, antimicrobial resistance, and serovar diversity of NTS in Ghana, which could inform strategies for controlling this important pathogen.

## Supplementary Information

Additional file 1: Table 1. Pooled estimate of all studies. Table 2. Influential analysis for all studies. Table 3. Linear regression test of funnel plot asymmetry for all studies. Table 4. Subgroup analysis for category. Table 5. Subgroup analysis for year groups. Table 6. Subgroup analysis for belts. Table 7. Subgroup analysis for type of sample. Table 8. Meta-regression analysis of year group effects on variability. Table 9. Meta-regression analysis of category effects on variability. Table 10. Meta-regression analysis of type of sample effects on variability. Table 11. Meta-regression analysis of year of publication effects on variability. Table 12. Meta-regression analysis of belts effects on variability. Table 13. Mixed-effects meta-regression: Assessing year group and category as moderators. Table 14. Mixed-effects meta-regression: Assessing year group and type of sample as moderators. Table 15. Mixed-effects meta-regression: Assessing year group and belts as moderators. Table 16. Mixed-effects meta-regression: Assessing type of sample and category as moderators. Table 17. Mixed-effects meta-regression: Assessing category and belts as moderators. Table 18. Mixed-effects meta-regression: Assessing year of publication and belts as moderators. Table 19. Mixed-effects meta-regression: Assessing year group, category, type of sample and belts as moderators.

Additional file 2: Table 1. Pooled estimate of Chloramphenicol resistance. Table 2. Influential analysis of chloramphenicol resistance. Table 3. Pooled estimate of co-trimoxazole resistance. Table 4. Influential analysis co-trimoxazole resistance. Table 5. Pooled estimate of tetracycline resistance. Table 6. Influential analysis of tetracycline resistance. Table 7. Pooled estimate of ciprofloxacin resistance. Table 8. Influential analysis ciprofloxacin resistance. Table 9. Pooled estimate of Cefuroxime resistance. Table 10. Influential analysis of cefuroxime resistance. Table 11. Pooled estimate of amoxicillin-clavulanate resistance. Table 12. Influential analysis of amoxicillin-clavulanate resistance. Table 13. Pooled estimate of amoxicillin-ampicillin resistance. Table 14. Pooled estimate of gentamicin resistance. Table 15. Influential analysis of gentamicin resistance. Table 16. Pooled estimate of nalidixic acid resistance. Table 17. Influential analysis of nalidixic acid resistance. Table 18. Pooled estimate of cefotaxime resistance. Table 19. Influential analysis cefotaxime resistance. Table 20. Pooled estimate of ampicillin resistance. Table 21. Influential analysis of ampicillin resistance. Table 22. Pooled estimate of sulfamethoxazole resistance. Table 23. Pooled estimate of trimethoprim resistance.

Additional file 3.

Additional file 4.

## Data Availability

No datasets were generated or analysed during the current study.

## Abbreviations

AAN: Asante Akyem North
HIV: Human Immunodeficiency Virus
iNTS: Invasive Non-typhoidal *Salmonella*
MDR: Multi drug resistance
NTS: Non-typhoidal *Salmonella*
OSF: Open Science Framework
PRISMA: Preferred Reporting Items for Systematic Reviews and Meta-analyses
SCD: Sickle cell disease
WHO: World Health Organization
