# Supplementary material for: Prevalence and antimicrobial resistance patterns of nontyphoidal Salmonella in Ghana: a systematic review and meta-analysis
Source: Trop Med Health. 2025 Jul 1;53:91. doi: 10.1186/s41182-025-00731-7 (PMC12210744; doi:10.1186/s41182-025-00731-7)
Supplement: Supplementary file 3 — Additional file 3. [file 41182_2025_731_MOESM3_ESM.docx]

**Table 1: Joanna Briggs Institute (JBI) Criteria for Prevalence Studies**

| **Study** | **Q1** | **Q2** | **Q3** | **Q4** | **Q5** | **Q6** | **Q7** | **Q8** | **Total score** | **Percentage** | **Study quality** |
| --- | --- | --- | --- | --- | --- | --- | --- | --- | --- | --- | --- |
| Acheampong et al. (2019) | Y | Y | Y | Y | Y | Y | Y | Y | 8 | 100% | High |
| Adjei et al. (2022) | Y | Y | N | Y | Y | Y | Y | Y | 7 | 87.5% | High |
| Adzitey et al. (2020) | Y | Y | Y | Y | Y | Y | Y | Y | 8 | 100% | High |
| Andoh et al. (2016) | Y | Y | Y | Y | Y | Y | Y | Y | 8 | 100% | High |
| Archer et al. (2023) | Y | Y | Y | Y | Y | Y | Y | Y | 8 | 100% | High |
| Dekker et al. (2015) | Y | Y | Y | Y | Y | Y | Y | Y | 8 | 100% | High |
| Dekker et al. (2018) | Y | U | Y | N | Y | Y | Y | Y | 7 | 87.5% | High |
| Dekker et al. (2019) | Y | N | Y | N | Y | Y | Y | Y | 6 | 75% | Moderate |
| Espinoza et al. (2016) | Y | Y | Y | Y | Y | Y | Y | Y | 8 | 100% | High |
| Djie Maletz et al. (2008) | Y | U | Y | Y | Y | Y | Y | Y | 7 | 87.5% | High |
| Donkor et al. (2023) | Y | Y | Y | Y | Y | Y | Y | Y | 8 | 100% | High |
| Eibach et al. (2016) | Y | U | Y | Y | Y | Y | Y | Y | 7 | 87.5% | High |
| Eibach et al. (2016b) | Y | U | Y | Y | Y | Y | Y | Y | 7 | 87.5% | High |
| Kumasi et al. (2011) | Y | Y | U | Y | Y | Y | Y | Y | 8 | 87.5% | High |
| Kunadu et al. (2018) | Y | Y | Y | Y | Y | Y | Y | Y | 8 | 100% | High |
| Kunadu et al. (2020) | Y | Y | Y | Y | Y | Y | Y | Y | 8 | 100% | High |
| Labi et al. (2014) | Y | Y | Y | Y | Y | Y | Y | Y | 8 | 100% | High |
| Mensah et al. (2002) | Y | U | Y | Y | Y | Y | Y | Y | 7 | 87.5% | High |
| Moirongo et al. (2018) | Y | U | Y | N | Y | Y | Y | Y | 6 | 75% | Moderate |
| Newman et al. (2011) | Y | U | Y | N | Y | U | U | Y | 4 | 50% | Moderate |
| Ofori et al. (2023) | Y | Y | Y | Y | Y | Y | Y | Y | 8 | 100% | High |
| Opintan et al. (2017) | Y | U | Y | Y | Y | Y | Y | Y | 7 | 87.5% | High |
| Saba et al. (2013) | Y | U | Y | N | U | Y | U | N | 4 | 50% | Moderate |
| Schwartz et al. (2010) | Y | U | Y | Y | Y | Y | Y | Y | 7 | 87.5% | High |
| Sothmann et al. (2015) | Y | U | Y | Y | Y | Y | Y | Y | 8 | 87.5% | High |
| Toy et al. (2019) | Y | U | U | Y | Y | Y | Y | Y | 6 | 75% | Moderate |

Key: Y= Yes; N=No; U=Unclear

**QUESTIONS**

| Q1. Was the sample frame appropriate to address the target population? |
| --- |
| Q2. Were study participants sampled in an appropriate way? |
| Q3. Was the sample size adequate?   \| Q4. Were the study subjects and the setting described in detail? \| \| --- \| \| Q5. Was the data analysis conducted with sufficient coverage of the identified sample? \| \| Q6. Were valid methods used for the identification of the condition? \| \| Q7. Was the condition measured in a standard, reliable way for all participants? \| \| Q8. Was there appropriate statistical analysis? \| \|  \| |

**Table 2: Study Characteristics of Included Articles**

| Study ID | Author | Year of Publication | Year Group | Study Type | Location | Belt | Period of Sample Collection | Study Subjects | Type of Sample | Number of Samples | Positive Samples | Method of Serovar Identification | Method of Antimicrobial Susceptibility Testing |
| --- | --- | --- | --- | --- | --- | --- | --- | --- | --- | --- | --- | --- | --- |
| ID001 | Acheampong et al. | 2019 | 2018-2023 | Retrospective | Kumasi | Middle | May 2016- January 2018 | Humans | Blood | 133 | 16 | Agglutination | Disk diffusion |
| ID001 | Acheampong et al. | 2019 | 2018-2023 | Retrospective | Kumasi | Middle | May 2016- January 2018 | Humans | Stool | 418 | 9 | Agglutination | Disk diffusion |
| ID001 | Acheampong et al. | 2019 | 2018-2023 | Retrospective | Kumasi | Middle | May 2016- January 2018 | Humans | Oropharynx | 558 | 1 | Agglutination | Disk diffusion |
| ID002 | Adjei et al. | 2022 | 2018-2023 | Not stated | Ashaiman | Lower | Not stated | Food | Raw beef | 80 | 6 | Agglutination | Not applicable |
| ID003 | Adzitey et al. | 2020 | 2018-2023 | Not stated | Tamale | Upper | Not stated | Food | Mixed | 225 | 16 | Whole Genome Sequencing | Disk diffusion |
| ID006 | Andoh et al. | 2016 | 2013-2017 | Cross-sectional | Accra and Kumasi | Mixed | August 2011-November 2012 | Animals | Skin neck | 30 | 3 | Agglutination | Disk diffusion |
| ID006 | Andoh et al. | 2016 | 2013-2017 | Cross-sectional | Accra and Kumasi | Mixed | August 2011-November 2012 | Environment | Mixed | 170 | 91 | Agglutination | Disk diffusion |
| ID007 | Archer et al | 2023 | 2018-2023 | Cross-sectional | Accra, Kumasi and Tamale | Mixed | October 2019-August 2020 | Food | Mixed | 384 | 21 | Whole Genome Sequencing | Broth microdilution |
| ID008 | Dekker et al. | 2015 | 2013-2017 | Not stated | Asante Akyem North | Middle | November 2009-November 2010 | Environment | Well water | 398 | 26 | Agglutination | Not stated |
| ID009 | Dekker et al. | 2018 | 2018-2023 | Not stated | Asante Akyem North | Middle | September 2007-November 2012 | Humans | Blood | 2720 | 165 | Agglutination | Disk diffusion |
| ID009 | Dekker et al. | 2018 | 2018-2023 | Not stated | Asante Akyem North | Middle | October-December 2009 | Environment | Mixed | 511 | 19 | Agglutination | Disk diffusion |
| ID010 | Dekker et al. | 2019 | 2018-2023 | Cross-sectional | Kumasi | Middle | May-December 2015 | Food | Chicken meat | 200 | 17 | MALDI-TOF | Disk diffusion |
| ID011 | Djie-Maletz et al. | 2008 | 2008-2012 | Not stated | Tamale | Upper | November 2005-January 2006 | Humans | Stool | 367 | 5 | Agglutination | Disk diffusion and broth microdilution |
| ID012 | Donkor et al. | 2023 | 2018-2023 | Cross-sectional | Accra, Kumasi, Ho, Effia Nkwanta, Koforidua, Maase-Offinso | Mixed | April-December 2021 | Humans | Blood | 334 | 18 | Not stated | Disk diffusion and BP Phoenix M50 analyzer |
| ID013 | Eibach et al. | 2016 | 2013-2017 | Not stated | Asante Akyem North | Middle | September 2007-December 2012 | Humans | Blood | 7172 | 215 | MALDI-TOF | VITEK 2 |
| ID014 | Eibach et al. | 2016 | 2013-2017 | Not stated | Asante Akyem North | Middle | September 2007-May 2012 | Humans | Blood | 5211 | 196 | PCR and agglutination | Disk diffusion |
| ID015 | Espinoza et al. | 2016 | 2013-2017 | Not stated | Asante Akyem North | Middle | March 2010-May 2012 | Humans | Blood | 2651 | 46 | Not stated | Not applicable |
| ID016 | Kumasi et al. | 2011 | 2008-2012 | Not stated | Barekese | Middle | January 2006-December 2007 | Environment | Water | Not stated | 2 | Not stated | Not applicable |
| ID018 | Kunadu et al. | 2018 | 2018-2023 | Not stated | Accra | Lower | July-October 2016 | Food | Mixed | 159 | 19 | Whole Genome Sequencing | VITEK 2 |
| ID019 | Kunadu et al. | 2020 | 2018-2023 | Not stated | Accra | Lower | Not stated | Environment | Mixed | 148 | 51 | Agglutination | Disk diffusion |
| ID020 | Labi et al. | 2014 | 2013-2017 | Retrospective | Accra | Lower | January 2010-December 2013 | Humans | Blood | 23708 | 115 | Agglutination | Disk diffusion |
| ID021 | Mensah et al. | 2002 |  | Not stated | Nima, Kotobabi, Pig Farm, Accra New Town# | Lower | Not stated | Food | Mixed | 511 | Not stated | Agglutination | Not applicable |
| ID022 | Moirongo et al. | 2018 | 2018-2023 | Not stated | Asante Akyem North | Middle | November 2013-March 2017 | Humans | Mixed | 1708 | 60 | Not stated | Disk diffusion |
| ID023 | Newman et al. | 2011 | 2008-2012 | Prospective quantitative | Not stated | Mixed | December 2002-December 2003 | Humans | Mixed | 5099 | 247 | Not stated | Disk diffusion |
| ID024 | Nielsen et al. | 2012 | 2008-2012 | Hospital-based | Asante Akyem North | Middle | September 2007-July 2009 | Humans | Blood | 1196 | 129 | Not stated | Disk diffusion |
| ID025 | Ofori et al. | 2023 | 2018-2023 | Cross-sectional | Kumasi and Ejisu | Middle | April 2019-November 2020 | Environment | Mixed | 1490 | 90 | Agglutination | Disk diffusion |
| ID026 | Opintan & Newman | 2017 | 2018-2023 | Not stated | Tamale, Kumasi, Sunyani, Sekondi, Cape Coast, Offinso, Eastern and Upper East | Mixed | June-November 2014 | Humans | Blood | 6351 | 4 | Not stated | Disk diffusion |
| ID027 | Saba et al. | 2013 | 2013-2017 | Not stated | Tamale | Upper | Jan-10 | Humans | Stool | 101 | 4 | Agglutination | Disk diffusion |
| ID028 | Schwartz et al. | 2010 | 2008-2012 | Not stated | Asante Akyem North | Middle | September 2007-July 2009 | Humans | Blood | 1032 | 128 | Not stated | Disk diffusion |
| ID029 | Sothmann et al. | 2015 | 2013-2017 | Not stated | Kumasi | Middle | January-December 2012 | Humans | Blood | 2306 | 24 | Not stated | Not applicable |
| ID030 | Toy et al. | 2019 | 2018-2023 | Not stated | Kumasi | Middle | January 2010-September 2012 | Humans | Blood | Not stated | 118 | Not stated | Disk diffusion |

**Table 3: Resistance Genes Identified in Studies**

| **Study ID** | Author | Year of Publication | Resistance Genes Identified |
| --- | --- | --- | --- |
| ID001 | Acheampong et al. | 2019 | gyrA, gyrB, parC, parE, qnrS |
| ID004 | Aldrich et al. | 2019 | gyrA, sul2, strA, strB, tetA |
| ID005 | Al-Emran et al. | 2016 | *gyrB* |
| ID007 | Archer et al. | 2023 | *gyrA, qnrB19, tetA, dfrA14 , sul2 , aph (3’’)-Ib, aph(6)-Id, dfrA1, sul1, sul2, catA1, aaaA1 , fosA7.2, aac6-Iy* |
| ID010 | Dekker et al. | 2019 | aacA4 , gyrA, parC, qnrB2, qnrB19 |
| ID014 | Eibach et al. | 2016 | gyrA, gyrB |
| ID017 | Kudirkierne et al. | 2018 | *blaTEM52-B, blaCTX-M15* |
| ID022 | Moirongo et al | 2018 | *gyrA* |
| ID031 | Van Puyvelde et al. | 2023 | gyrA, gyrB |

**Table** **1:** **Serovar** **Distribution** **in** **NTS** **Isolated** **from** **Human,** **Food,** **Environmental** **and** **Animal** **Sources**

| **No** | **Serovar** | **Humans** | **Food** | **Environment** | **Animals** |
| --- | --- | --- | --- | --- | --- |
| 1 | *S*. Rostock | 1 | 0 | 0 | 0 |
| 2 | *S*. Heidelberg | 1 | 0 | 0 | 0 |
| 3 | *S*. Urbana | 1 | 0 | 0 | 0 |
| 4 | *S*. Ouakum | 1 | 2 | 0 | 0 |
| 5 | *S*. Colindale | 1 | 0 | 9 | 0 |
| 6 | *S*. Virchow | 1 | 0 | 9 | 0 |
| 7 | *S*. Poona | 1 | 4 | 6 | 0 |
| 8 | *S*. Muenster | 1 | 10 | 9 | 0 |
| 9 | *S*. Stanleyville | 3 | 0 | 7 | 0 |
| 10 | *S*. Dublin | 28 | 0 | 0 | 0 |
| 11 | *S*. Enteritidis | 37 | 4 | 19 | 0 |
| 12 | *S*. Typhimurium | 256 | 6 | 16 | 0 |
| 13 | *S*. Infantis | 0 | 1 | 10 | 0 |
| 14 | *S*. Africana | 0 | 1 | 0 | 0 |
| 15 | *S*. Lagos | 0 | 2 | 0 | 0 |
| 16 | *S*. Ajiobo | 0 | 2 | 1 | 0 |
| 17 | *S*. Agama | 0 | 3 | 1 | 0 |
| 18 | *S*. Kaapstad | 0 | 4 | 0 | 0 |
| 19 | *S*. Kentucky | 0 | 5 | 22 | 0 |
| 20 | *S*. Chester | 0 | 5 | 2 | 0 |
| 21 | *S*. Hato | 0 | 6 | 0 | 0 |
| 22 | *S*. Hader | 0 | 7 | 0 | 0 |
| 23 | *S*. Legon | 0 | 9 | 0 | 0 |
| 24 | *S*. Ituri | 0 | 0 | 1 | 0 |
| 25 | *S*. Corvallis | 0 | 0 | 1 | 0 |
| 26 | *S*. Georgia | 0 | 0 | 1 | 0 |
| 27 | *S*. Kingston | 0 | 0 | 1 | 0 |
| 28 | *S*. Mim | 0 | 0 | 1 | 0 |
| 29 | *S*. Pasing | 0 | 0 | 1 | 0 |
| 30 | *S*. Rovaniemi | 0 | 0 | 1 | 0 |
| 31 | *S*. Gaminara | 0 | 0 | 1 | 0 |
| 32 | *S*. Konongo | 0 | 0 | 1 | 0 |
| 33 | *S*. Redhill | 0 | 0 | 1 | 0 |
| 34 | *S*. Saarbruecken | 0 | 0 | 1 | 0 |
| 35 | *S*. Adelaide | 0 | 0 | 1 | 0 |
| 36 | *S*. Westhampton | 0 | 0 | 1 | 0 |
| 37 | *S*. Alachua | 0 | 0 | 1 | 0 |
| 38 | *S*. Honelis | 0 | 0 | 1 | 0 |
| 39 | *S*. Saphra | 0 | 0 | 1 | 0 |
| 40 | *S*. Mundonobo | 0 | 0 | 1 | 0 |
| 41 | *S*. Elisabethville | 0 | 0 | 1 | 0 |
| 42 | *S*. Durban | 0 | 0 | 1 | 0 |
| 43 | *S*. Mississippi | 0 | 0 | 1 | 0 |
| 44 | *S*. Wien | 0 | 0 | 1 | 0 |
| 45 | *S*. Wagenia | 0 | 0 | 1 | 0 |
| 46 | *S*. Lexington | 0 | 0 | 1 | 0 |
| 47 | *S*. Aschersleben | 0 | 0 | 1 | 0 |
| 48 | *S*. Oskarshamn | 0 | 0 | 1 | 0 |
| 49 | *S*. Westphalia | 0 | 0 | 2 | 0 |
| 50 | *S*. Montevideo | 0 | 0 | 2 | 0 |
| 51 | *S*. Epinay | 0 | 0 | 2 | 0 |
| 52 | *S*. Illala | 0 | 0 | 2 | 0 |
| 53 | *S*. Give | 0 | 0 | 2 | 0 |
| 54 | *S*. Pramiso | 0 | 0 | 3 | 0 |
| 55 | *S*. Derby | 0 | 0 | 3 | 3 |
| 56 | S. Reading | 0 | 0 | 3 | 0 |
| 57 | *S*. Santander | 0 | 0 | 4 | 0 |
| 58 | *S*. Seftenberg | 0 | 0 | 4 | 0 |
| 59 | *S*. Yovokome | 0 | 0 | 4 | 0 |
| 60 | *S*. Haifa | 0 | 0 | 5 | 0 |
| 61 | *S*. Agona | 0 | 0 | 7 | 0 |
| 62 | *S*. Bochum | 0 | 0 | 7 | 0 |
| 63 | *S*. Cairina | 0 | 0 | 8 | 0 |
| 64 | *S*. Newport | 0 | 0 | 8 | 0 |
| 65 | *S*. Rubislaw | 0 | 0 | 37 | 0 |
| 66 | *S*. Tamale | 0 | 0 | 11 | 0 |
| 67 | *S*. Nima | 0 | 0 | 16 | 0 |
| 68 | *S*. Lexington | 0 | 0 | 1 | 0 |
| 69 | *S*. Duisburg | 0 | 0 | 10 | 0 |

**Table** **2:** **Characteristic** **of** **Included** **Studies** **of** **Non-Typhoidal** ***Salmonella*** **Prevalence** **in** **Ghana** **(Meta-analysis)**

|  | Study | Year  of Publication | Year Group | Belt | Type of study | Category | Period of Sample Collection | Type of  Sample | Sample Size | Positive  Samples | NTS/  Serovars isolated |
| --- | --- | --- | --- | --- | --- | --- | --- | --- | --- | --- | --- |
| 1 | Acheampong et al. (2019) | 2019 | 2018-2023 | Middle | Retrospective | Humans | May 2016- January 2018 | Blood | 133 | 16 | S. Typhimurium  S. Enteritidis |
| 2 | Acheampong et al. (2019) | 2019 | 2018-2023 | Middle | Retrospective | Humans | May 2016- January 2018 | Stool | 418 | 9 | S. Typhimurium  S. Enteritidis |
| 5 | Adzitey et al. (2020) | 2020 | 2018-2023 | Upper | NS | Food |  | Mixed | 225 | 16 | S. Kaapstad S. Lagos S. Ouakum S. Infantis S. Africana S. Hato  II 13,22:z |
| 7 | Andoh et al. (2016) | 2016 | 2013-2017 | Mixed | Cross-sectional | Environment | August 2011-November 2012 | Mixed | 170 | 91 | S. Typhimurium  S. Ituri   S. Oskarshamn  S. Stanleyville  S. Duisburg  S. Bochum  S. Poona  S. Rubislaw  S. Haifa  S. Cairina  S. Virchow  S. Muenster  S. Enteritidis  S. Nima  S. Kentucky |
| 10 | Dekker et al. (2018) | 2018 | 2018-2023 | Middle | NS | Humans | September 2007- November 2012 | Blood | 2720 | 165 | S. Typhimurium  S. Enteritidis   S. Dublin  S. Heidelberg S. Rostock S. Virchow S. Stanleyville |
| 11 | Dekker et al. (2018) | 2018 | 2018-2023 | Middle | NS | Environment | October 2009 -December 2009 | Mixed | 511 | 19 | S. Ajiobo  S. Colindale S. Corvallis  S. Duisburg  S. Georgia  S. Kingston  S. Mim  S. Poona  S. Pramiso   S. Rovaniemi  S. Pasing  S. Rubislaw  S. Santander  S. Stanleyville |
| 13 | Djie-Maletz et al. (2008) | 2008 | 2008-2012 | Upper | NS | Humans | November 2005-January 2006 | Stool | 367 | 5 | Salmonella enterica Group 2 S. Typhimurium S. Colindale |
| 14 | Donkor et al. (2023) | 2023 | 2018-2023 | Mixed | Cross-sectional | Humans | April- December 2021 | Blood | 334 | 18 | NS |
| 15 | Eibach et al. (2016) | 2016 | 2013-2017 | Middle | NS | Humans | September 2007- July 2009  January 2010- December 2012 | Blood | 7172 | 215 | NS |
| 16 | Eibach et al. (2016b) | 2016 | 2013-2017 | Middle | NS | Humans | September 2007- May 2012 | Blood | 5211 | 196 | S. Typhimurium  S. Enteritidis   S. Dublin S. Virchow S. Stanleyville S. Poona S. Muenster |
| 17 | Espinoza et al. (2016) | 2016 | 2013-2017 | Middle | NS | Humans | March 2010- May 2012 | Blood | 2651 | 46 | NS |
| 18 | Kunadu et al. (2018) | 2018 | 2018-2023 | Lower | NS | Food | July-October 2016 | Mixed | 159 | 19 | S. Muenster S. Legon |
| 19 | Kunadu et al. (2020) | 2020 | 2018-2023 | Lower | NS | Environment | NS | Mixed | 148 | 51 | S. Agona  S. Enteritidis S. Infantis  S. Mississipi  S. Newport  S. Typhimurium  S. Seftenberg S. Adelaide S. Westhampton |
| 20 | Labi et al. (2014) | 2014 | 2013-2017 | Lower | Retrospective | Humans | January 2010- December 2013 | Blood | 23708 | 115 | NS |
| 21 | Moirongo et al. (2018) | 2018 | 2018-2023 | Middle | NS | Humans | November 2013-March 2017 | Mixed | 1708 | 60 | NS |
| 22 | Newman et al. (2011) | 2011 | 2008-2012 | Mixed | Prospective quantitative | Humans | December 2002-December 2003 | Mixed | 5099 | 247 | NS |
| 23 | Nielsen et al. (2012) | 2012 | 2008-2012 | Middle | Hospital-based | Humans | September 2007-July 2009 | Blood | 1196 | 129 | NS |
| 24 | Ofori et al. (2023) | 2023 | 2018-2023 | Middle | Cross-sectional | Environment | April 2019-November 2020 | Mixed | 1490 | 90 | S. Rubislaw  S. Tamale   S. Kentucky   S. Bochum   S. Yovokome  S. Agona   S. Reading   S. Westphalia   S. Montevideo   S. Chester   S. Epinay  S. Ilala S. Agama  S. Alachua   S. Duisburg   S. Give   S. Honelis S. Poona  S. Mundonobo  S. Saphra  Serologically Rough  S. Typhimurium  S. Durban  S. Elisabethville  S. Redhill  S. Konongo  S. Aschersleben  S. Gaminara  S. Wagenia  S. Wien  S. Lexington  diarizonae Ssp. IIIb salamae Ssp. II |
| 25 | Opintan and Newman (2017) | 2017 | 2013-2017 | Mixed | NS | Humans | June–November 2014 | Blood | 6351 | 4 | NS |
| 26 | Saba et al. (2013) | 2013 | 2013-2017 | Upper | NS | Humans | January 2010 | Stool | 101 | 4 | S. Urbana S. Ouakam S. Stanleyville  S. Senftenberg |
| 27 | Schwartz et al. (2010) | 2010 | 2008-2012 | Middle | NS | Humans | September 2007-July 2009 | Blood | 1032 | 128 | NS |
| 28 | Sothmann et al. (2015) | 2015 | 2013-2017 | Middle | NS | Humans | January-December 2012 | Blood | 2306 | 24 | NS |

**Table** **4:** **Pooled** **antibiotic** **resistance** **levels** **(meta-analysis)**

| No | Study | Subject | Type of samples | Chloramphenicol_num | Chloramphenicol_total | Co-trimoxazole_num | Co-trimoxazole_total | Tetracycline_num | Tetracycline_total | Ciprofloxacin_num | Ciprofloxacin_total | Cefuroxime_num | Cefuroxime_total | Cefuroxime | Amoxicillin-clavulanate_num | Amoxicillin-clavulanate_total | Amoxicillin/ampicillin_num | Amoxicillin/ampicillin_total | Gentamicin_num | Gentamicin_total | Nalidixic acid_num | Nalidixic acid_total | Cefotaxime_num | Cefotaxime_total | Ampicillin_num | Ampicillin_total | Sulfamethoxazole_num | Sulfamethoxazole_total | Trimethoprim_num | Trimethoprim_total | Trimethoprim/sulfamethoxazole_num | Trimethoprim/sulfamethoxazole_total |
| --- | --- | --- | --- | --- | --- | --- | --- | --- | --- | --- | --- | --- | --- | --- | --- | --- | --- | --- | --- | --- | --- | --- | --- | --- | --- | --- | --- | --- | --- | --- | --- | --- |
| 1 | Schwartz et al. (2010) | Humans | Blood | 23 | 127 | 23 | 98 | 81 | 91 | 127 | 127 | 59 | 127 | 46.46 | 29 | 113 | 19 | 123 | 90 | 127 |  |  |  |  |  |  |  |  |  |  |  |  |
| 2 | Nielsen et al. (2012) | Humans | Blood | 105 | 127 | 76 | 98 | 8 | 91 |  |  |  |  |  | 86 | 114 | 105 | 123 |  |  | 2 | 127 |  |  |  |  |  |  |  |  |  |  |
| 3 | Labi et al. (2014) | Humans | Blood |  |  |  |  |  |  | 1 | 135 |  |  |  |  |  |  |  | 9 | 93 |  |  | 6 | 99 |  |  |  |  |  |  |  |  |
| 4 | Dekker et al. (2015) | Environment | Well water |  |  |  |  | 1 | 26 |  |  |  |  |  |  |  |  |  |  |  |  |  |  |  | 2 | 26 |  |  |  |  |  |  |
| 5 | Andoh et al. (2016) | Animal and Environment | Faecal sock Skin neck Dust Feed Drinking water | 4 | 94 |  |  | 46 | 94 | 37 | 94 |  |  |  | 9 | 94 |  |  | 9 | 94 | 51 | 94 |  |  | 14 | 94 | 24 | 94 | 18 | 94 |  |  |
| 6 | Eibach et al. (2016) | Humans | Blood | 137 | 196 |  |  |  |  | 14 | 215 |  |  |  |  |  |  |  |  |  |  |  |  |  | 149 | 196 | 137 | 196 |  |  |  |  |
| 7 | Opintan et al. (2017) | Humans | Blood | 3 | 4 |  |  | 3 | 4 |  |  | 3 | 4 | 75 |  |  |  |  | 3 | 4 |  |  | 3 | 4 | 3 | 4 |  |  |  |  | 3 | 4 |
| 8 | Dekker et al. (2019) | Food | Chicken meat | 1 | 16 |  |  | 16 | 16 | 10 | 16 |  |  |  | 5 | 16 |  |  |  |  |  |  |  |  | 9 | 16 |  |  |  |  | 11 | 16 |
| 9 | Kunadu et al. (2020) | Environment |  | 2 | 51 |  |  | 21 | 51 | 11 | 51 | 3 | 51 | 5.88 | 21 | 51 |  |  | 5 | 51 |  |  | 2 | 51 | 11 | 51 |  |  |  |  | 28 | 51 |
| 10 | Archer et al. (2023) | Food | Eggshells Egg content | 2 | 16 |  |  | 8 | 16 | 15 | 16 |  |  |  |  |  |  |  |  |  | 15 | 16 |  |  |  |  | 8 | 16 | 8 | 16 |  |  |
| 11 | Donkor et al. (2023) | Humans | Blood | 5 | 18 |  |  | 6 | 18 | 5 | 18 |  |  |  |  |  |  |  | 4 | 18 | 3 | 18 | 7 | 18 | 6 | 18 |  |  |  |  | 6 | 18 |
| 12 | Ofori et al. (2023) | Environment | Pooled faecal Dust Soil |  |  |  |  |  |  | 11 | 90 |  |  |  |  |  |  |  |  |  |  |  |  |  |  |  |  |  |  |  |  |  |


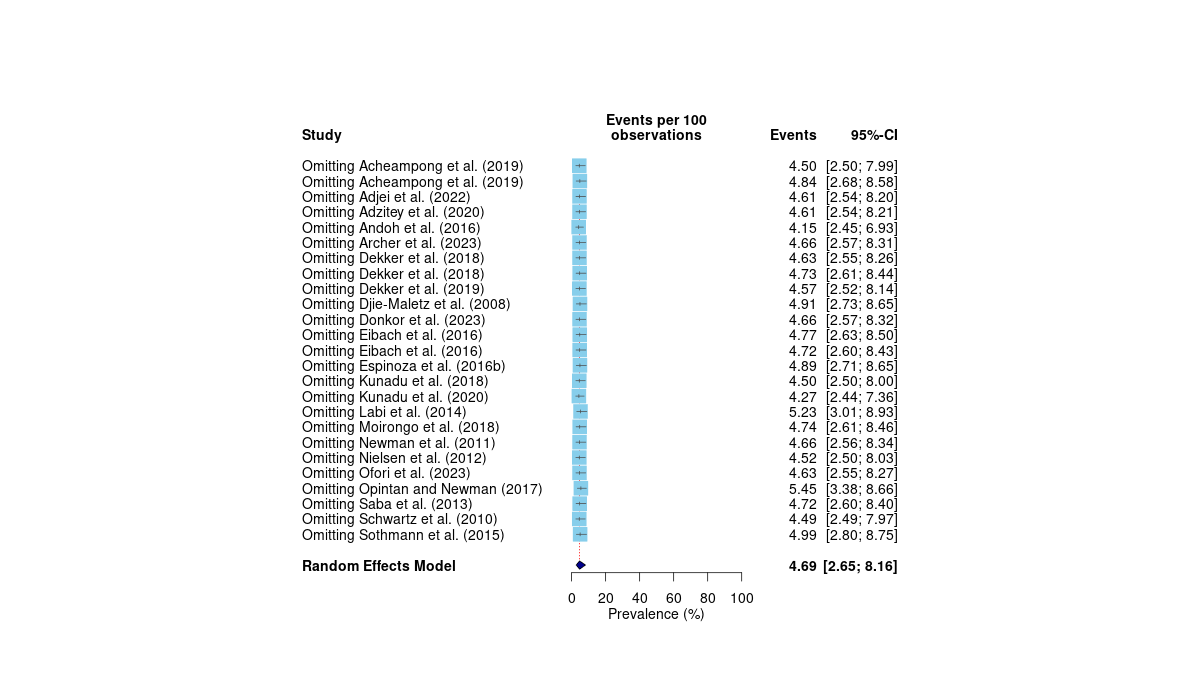
**Supplementary** **Figures**

SF1: Influential analysis for pooled prevalence of NTS in Ghana


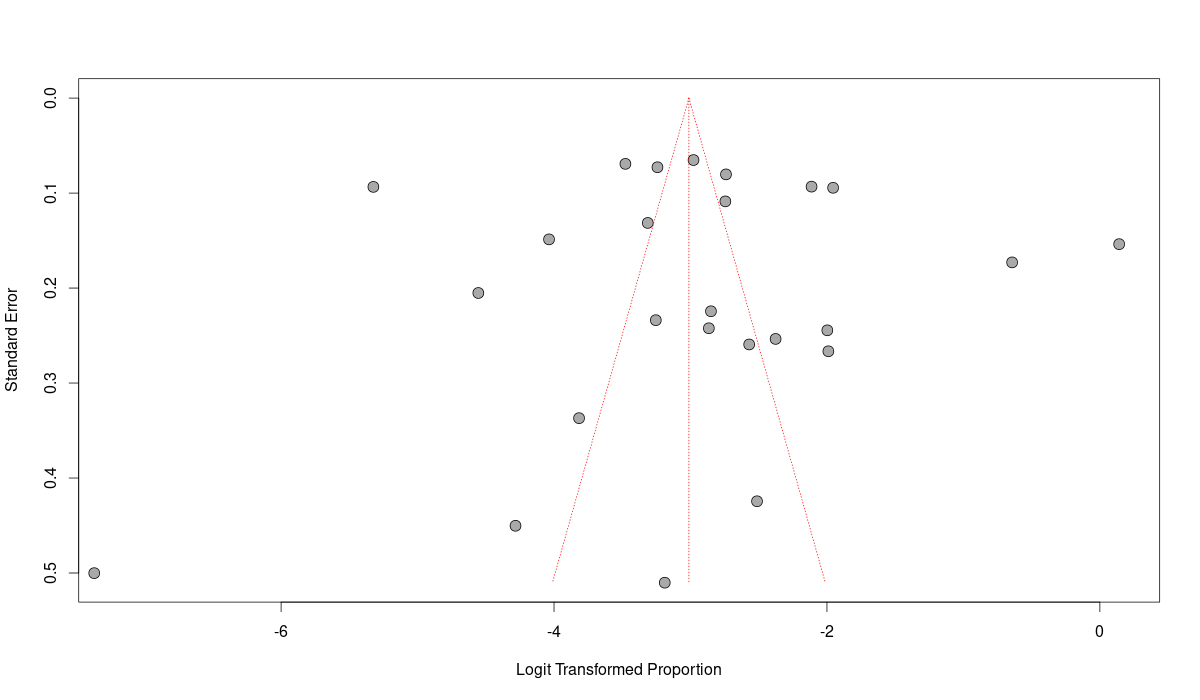


SF2: Funnel plot asymmetry to determine bias in prevalence of NTS in Ghana


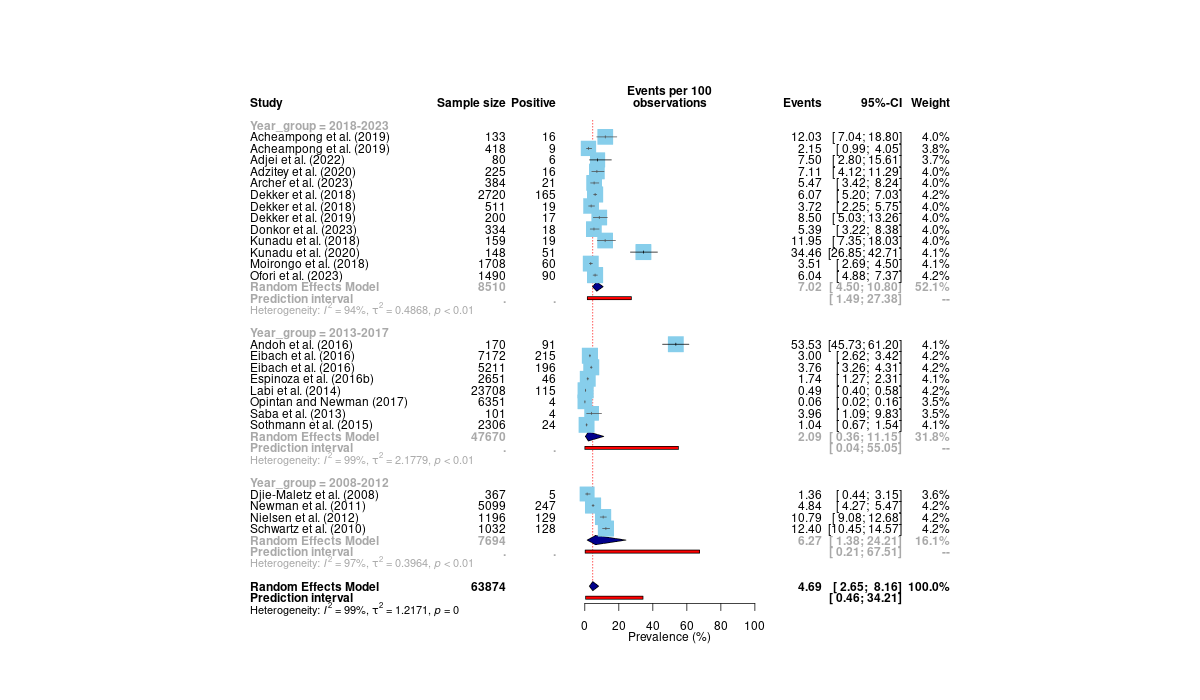


SF3: Figure of subgroup analysis based on year groupings.


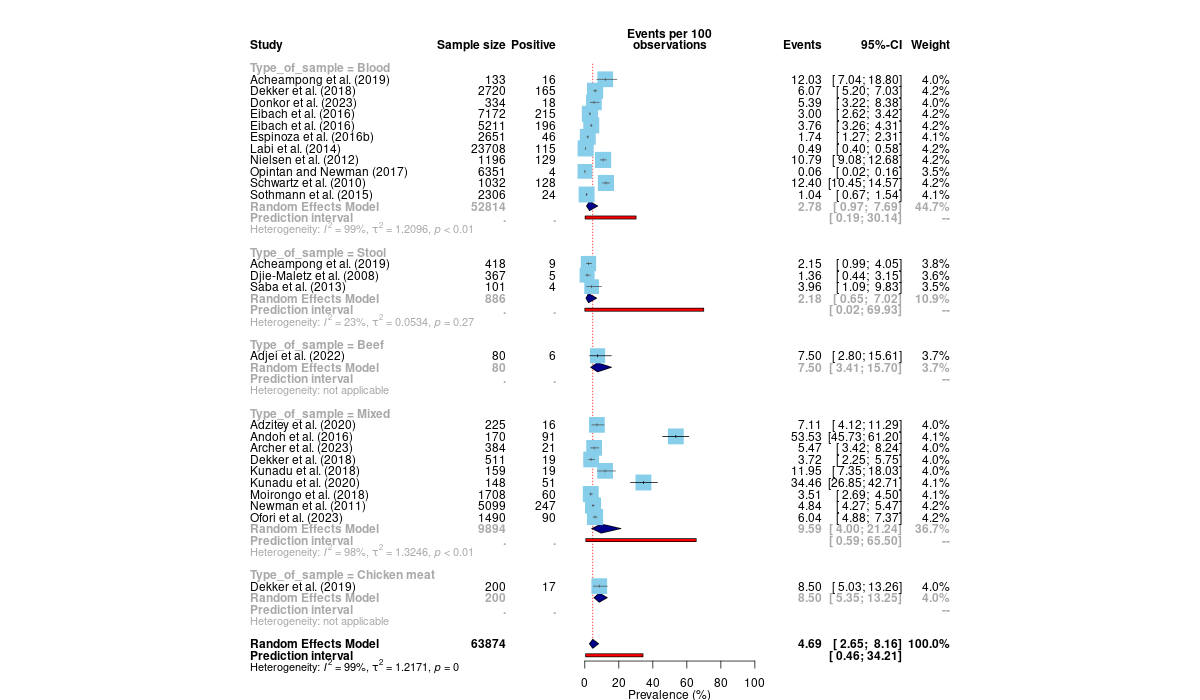
SF4: Figure of subgroup analysis based on samples.


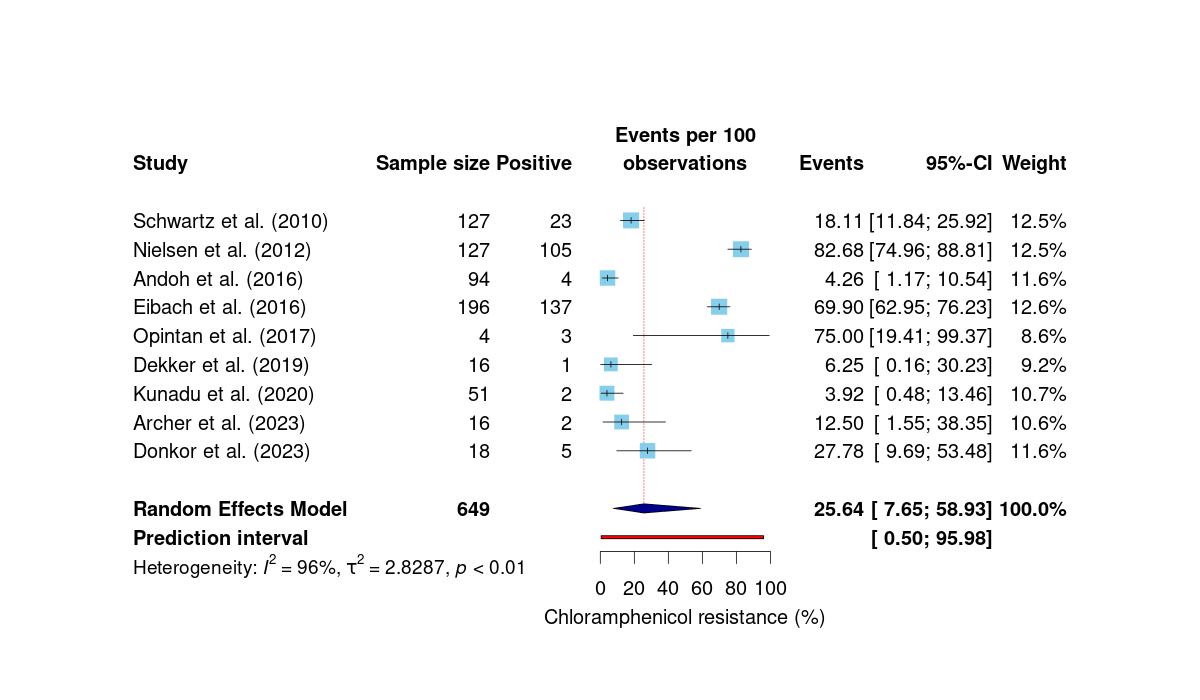


SF5: Figure of pooled resistance of chloramphenicol.


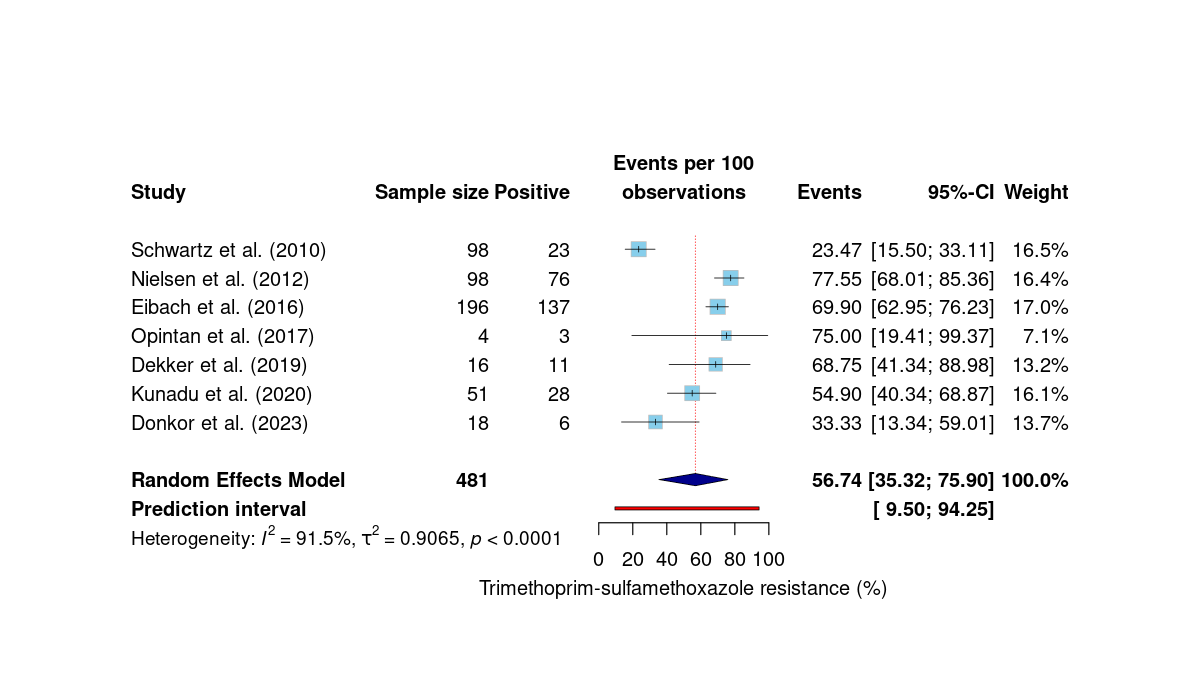


SF6: Figure of pooled resistance of trimethoprim-sulfamethoxazole.


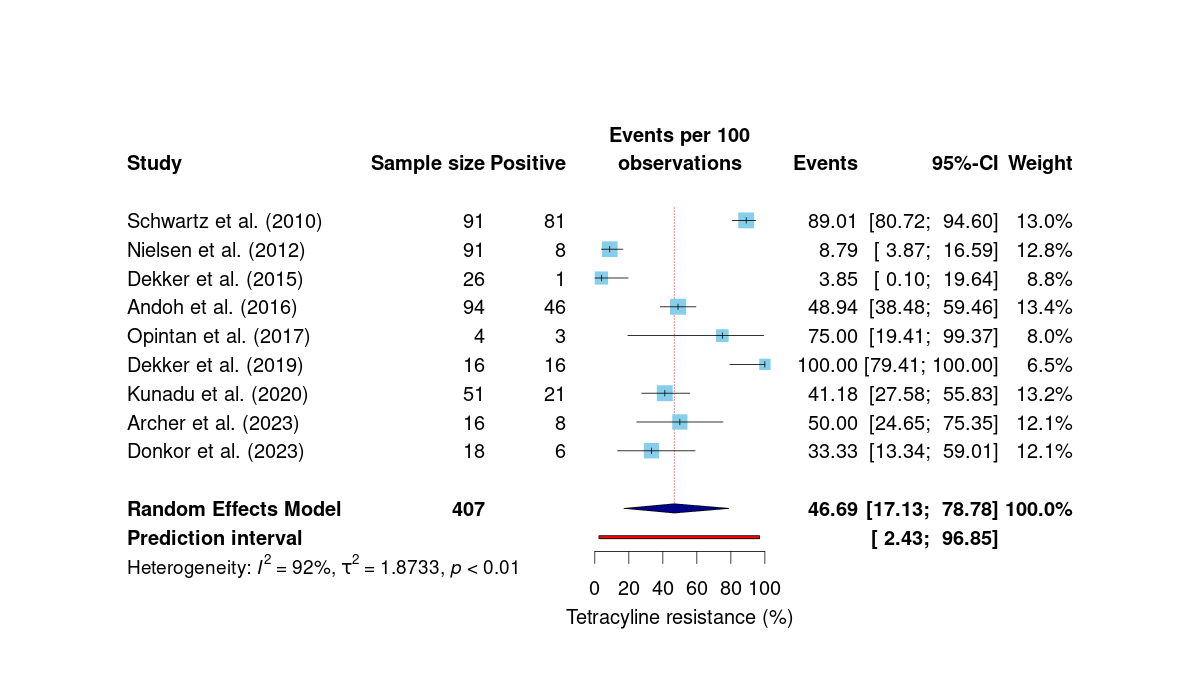


SF7: Figure of pooled resistance of tetracycline.


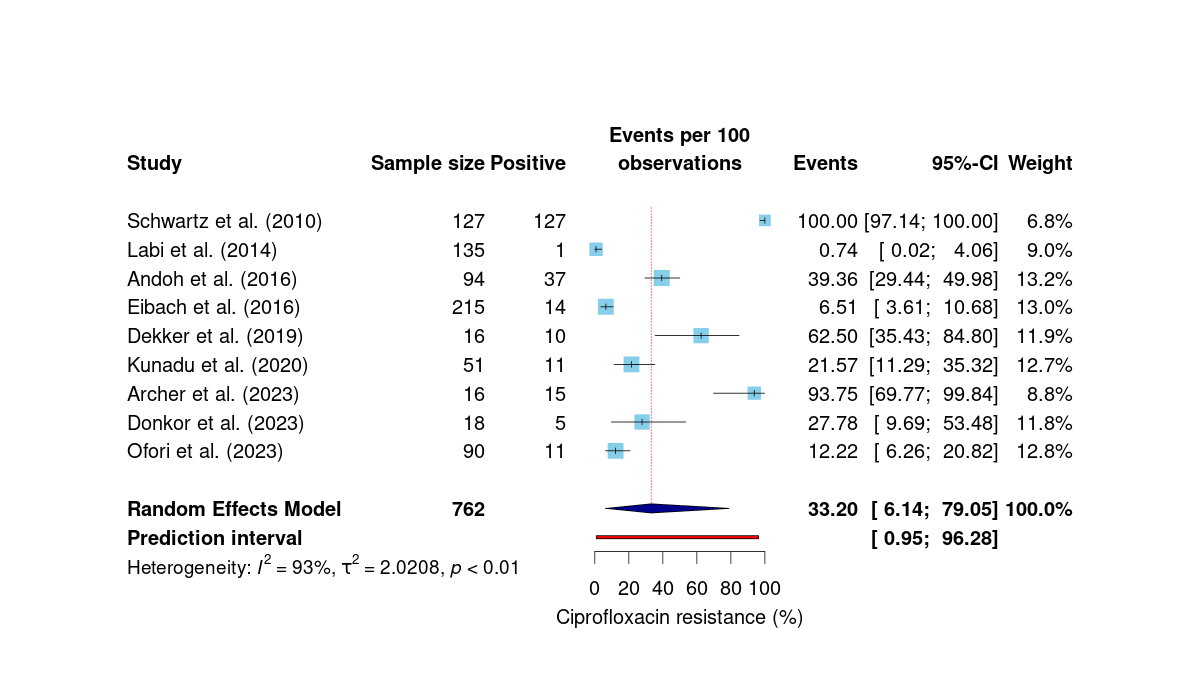


SF8: Figure of pooled resistance of ciprofloxacin.


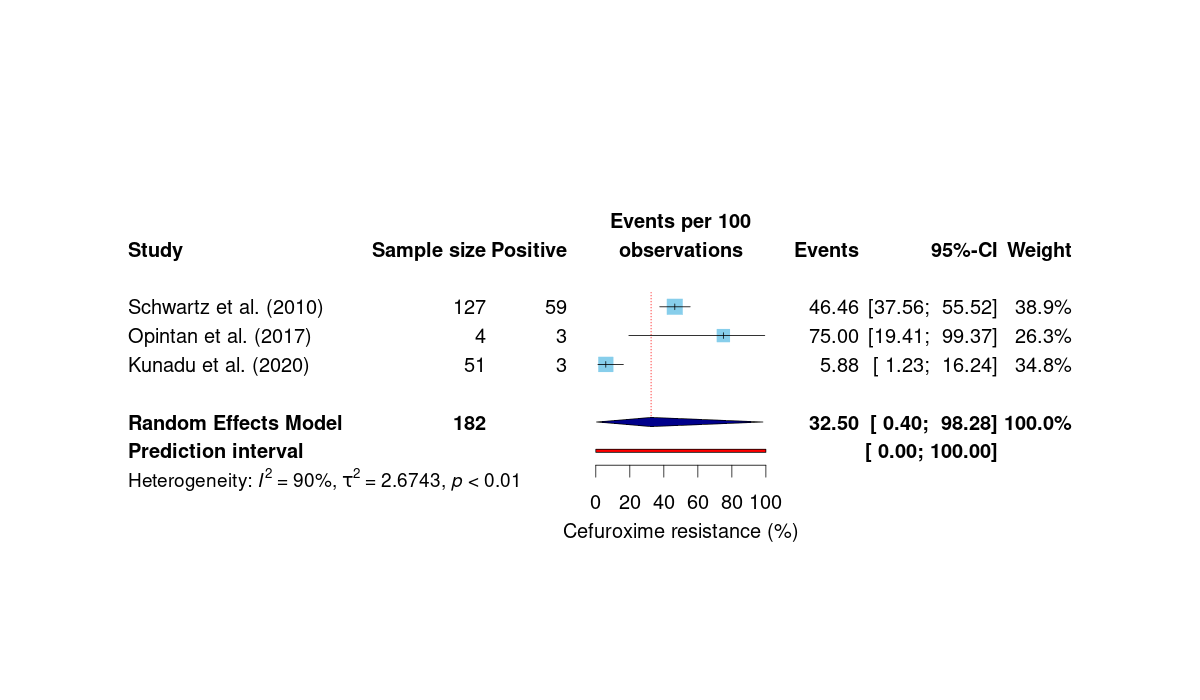


SF9: Figure of pooled resistance of cefuroxime


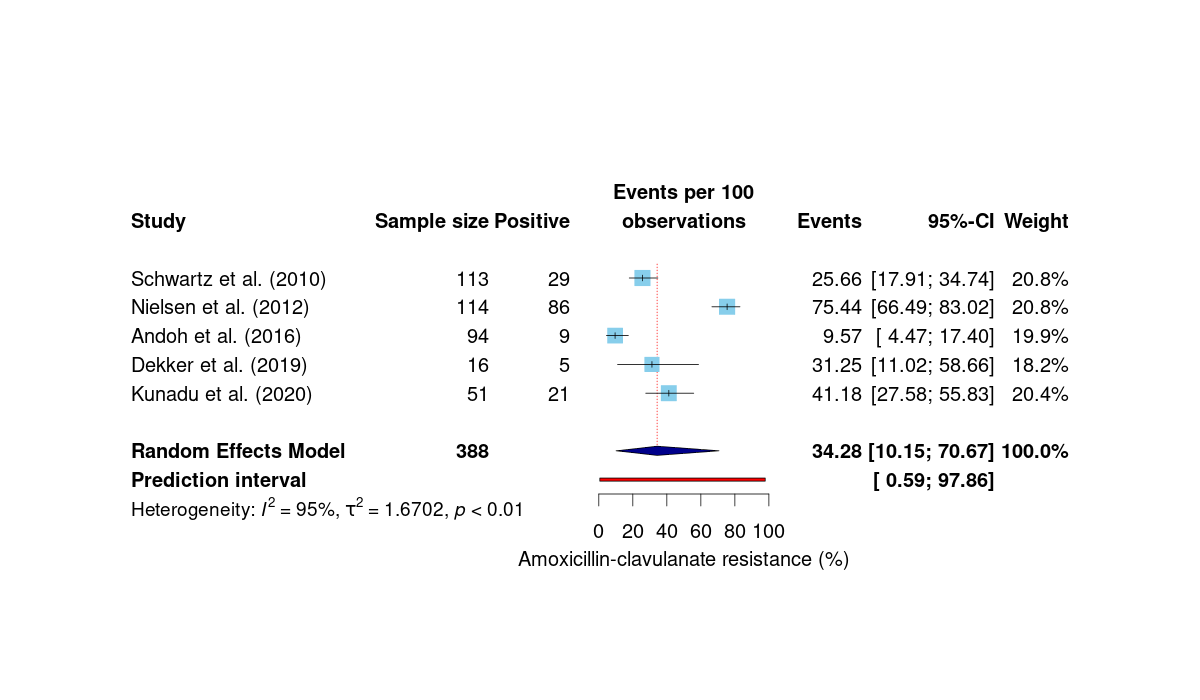


SF10: Figure of pooled resistance of amoxicillin-clavulanate


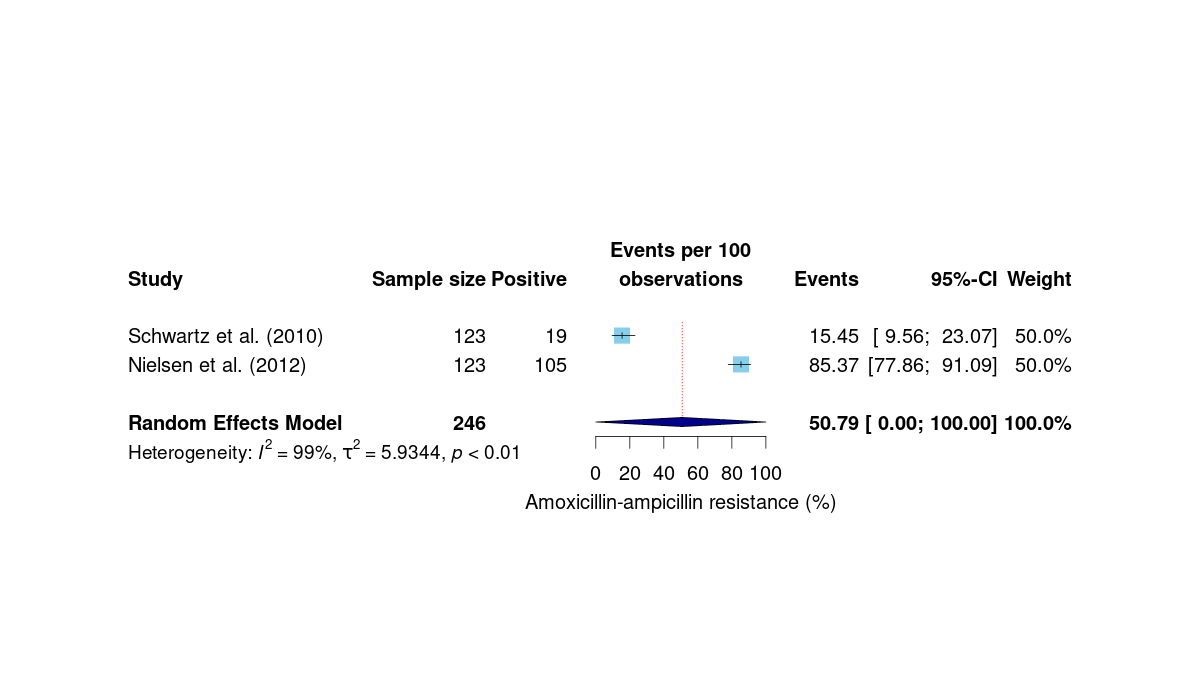


SF11: Figure of pooled resistance of amoxicillin/ampicillin


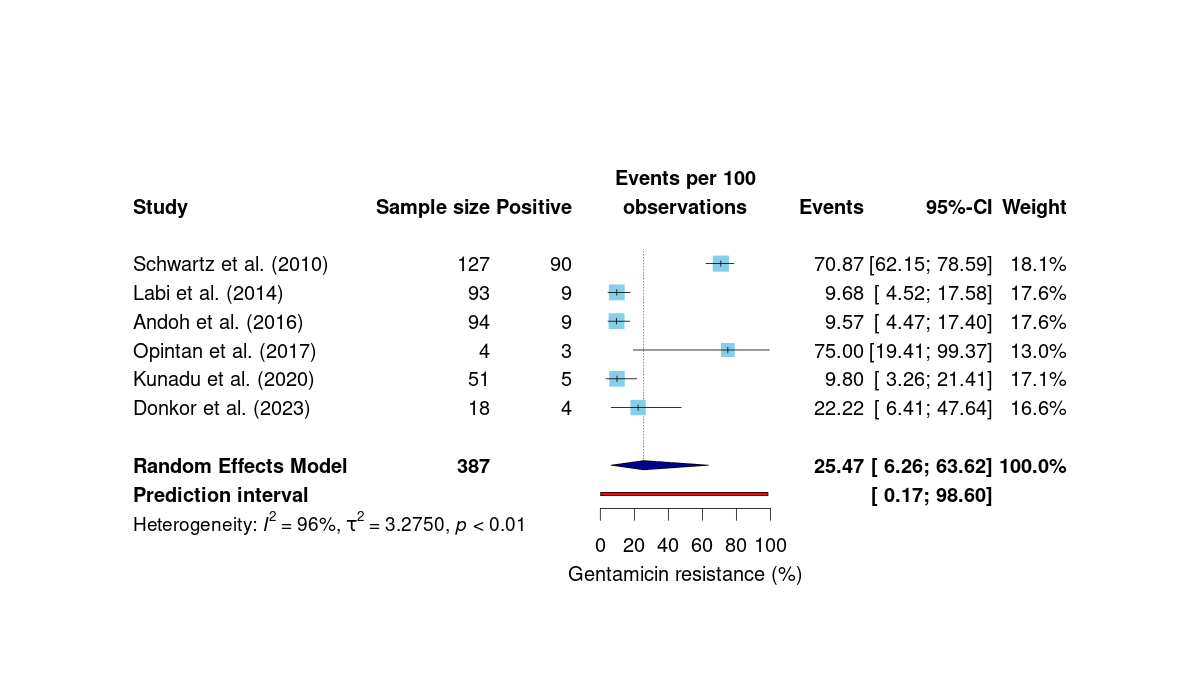
SF12: Figure of pooled resistance of gentamicin


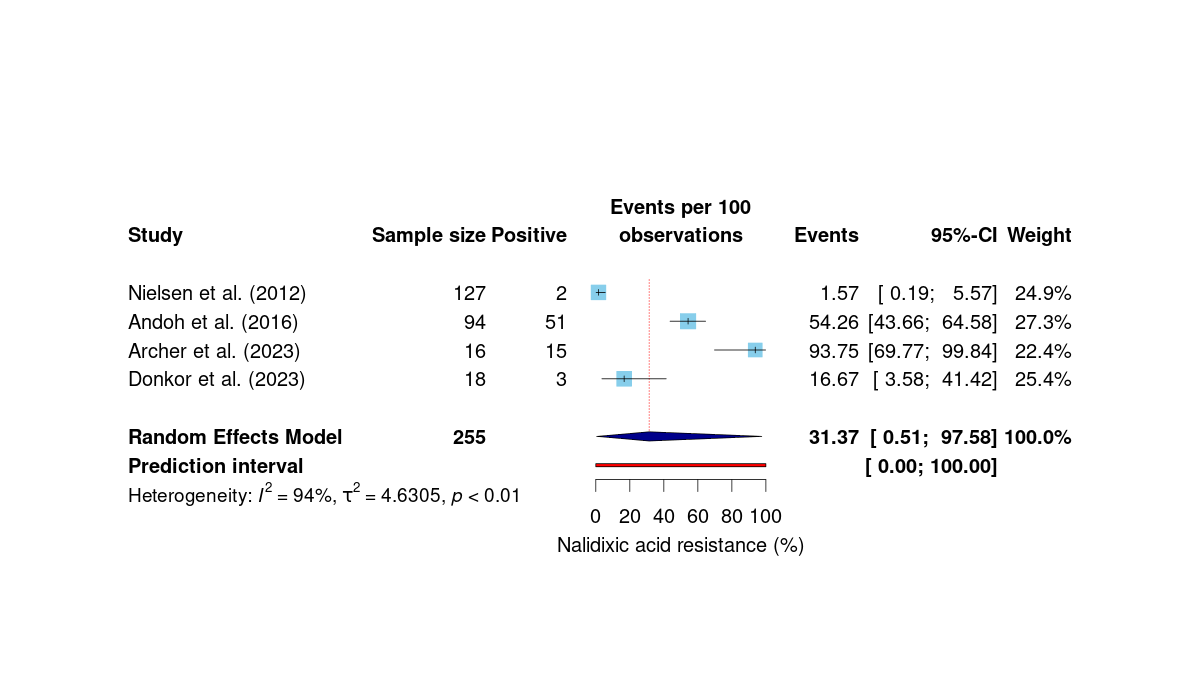
SF13: Figure of pooled resistance of nalidixic acid


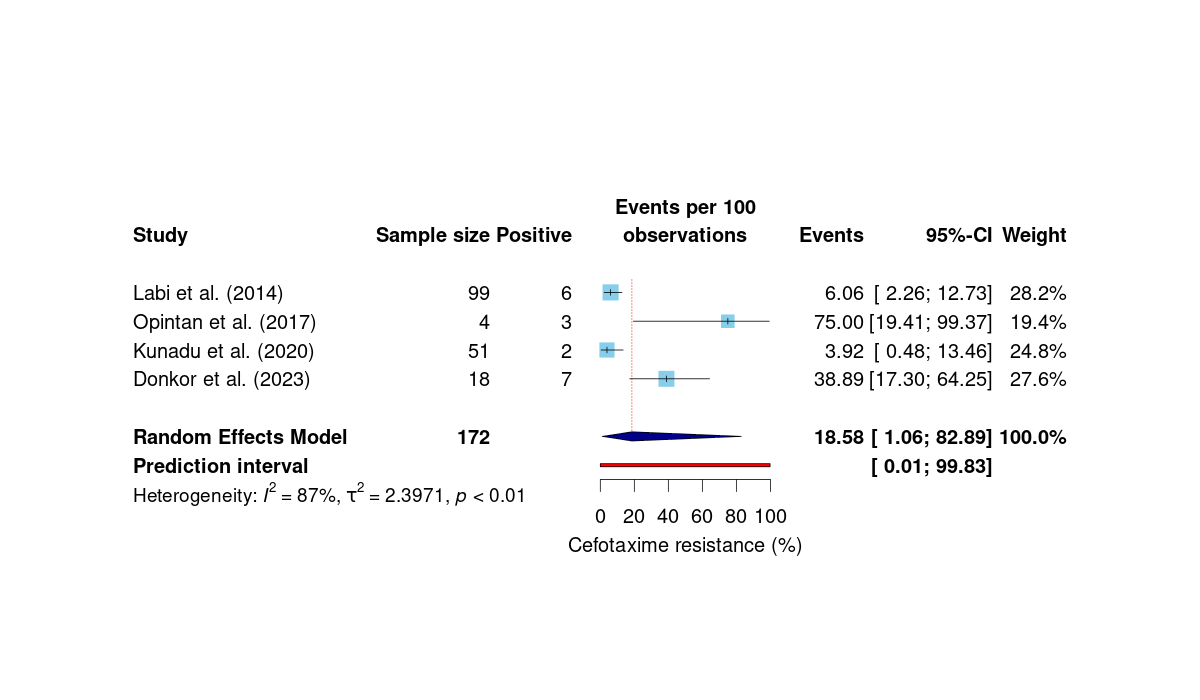


SF14: Figure of pooled resistance of cefotaxime


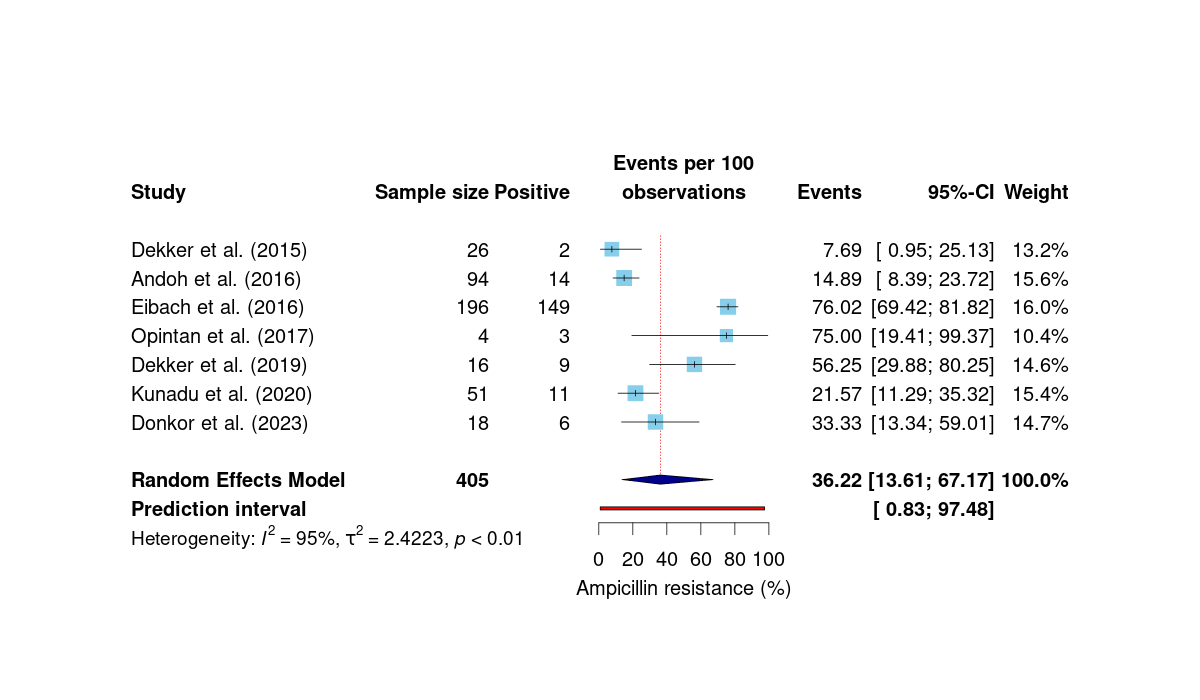


SF15: Figure of pooled resistance of ampicillin


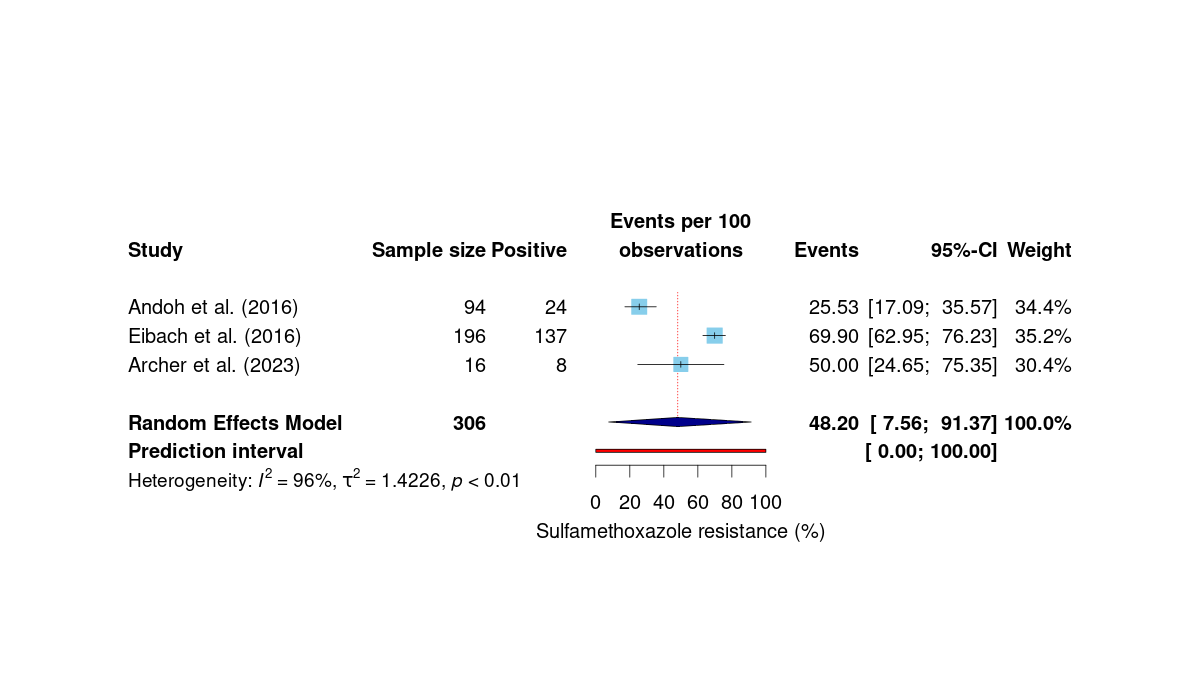


SF16: Figure of pooled resistance of sulfamethoxazole


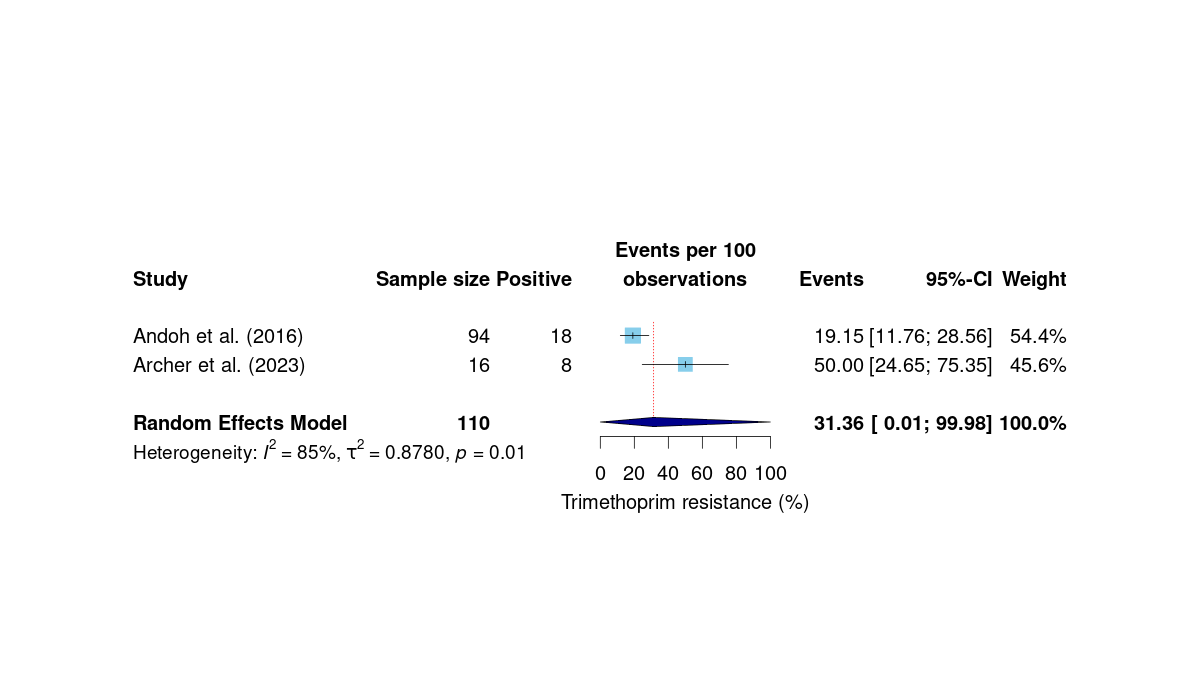


SF17: Figure of pooled resistance of trimethoprim


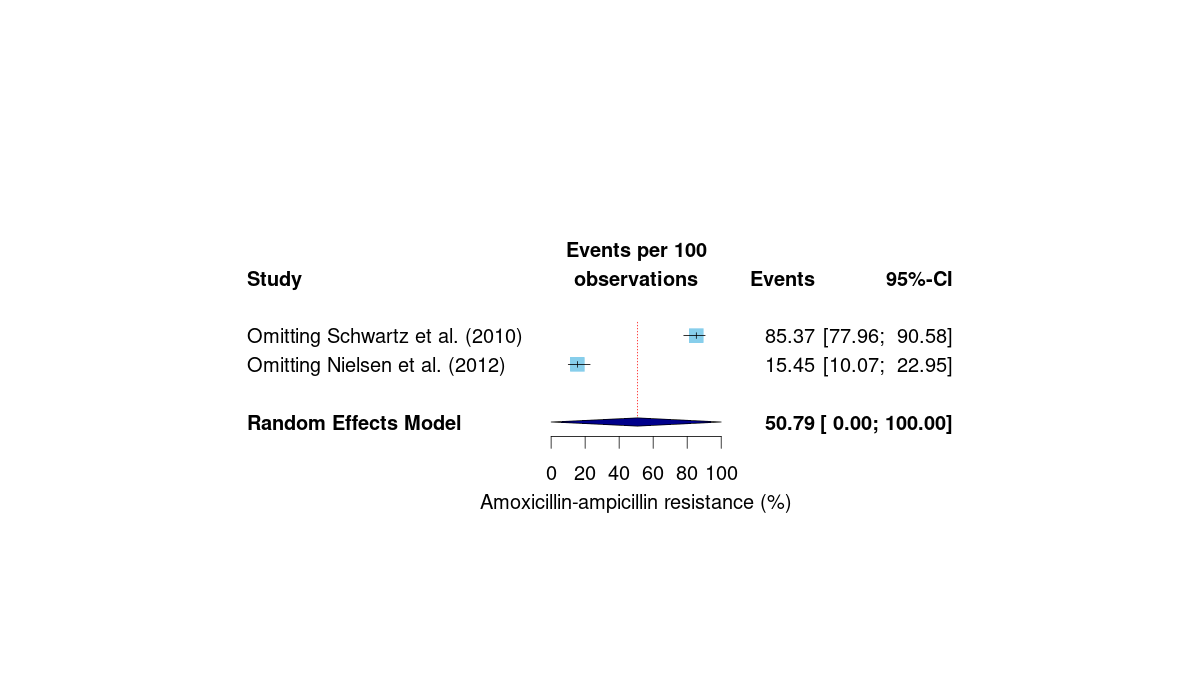
SF 18: Figure of influential analysis for amoxicillin-ampicillin


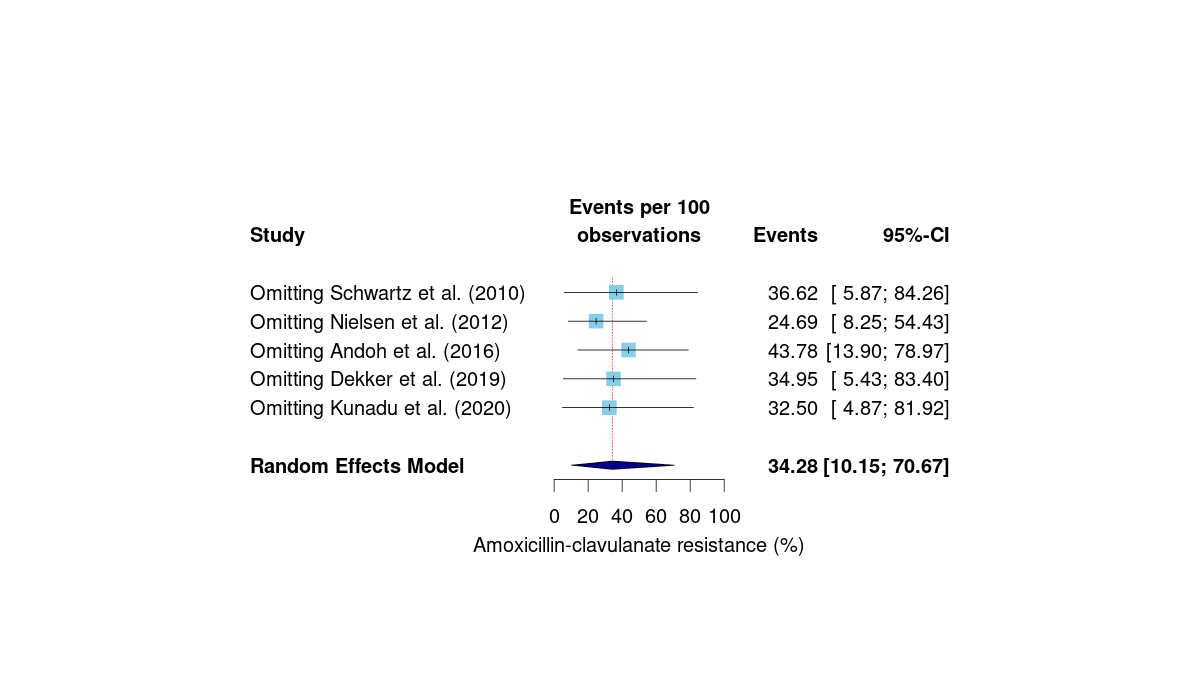
SF 19: Figure of influential analysis for amoxicillin-clavulanate


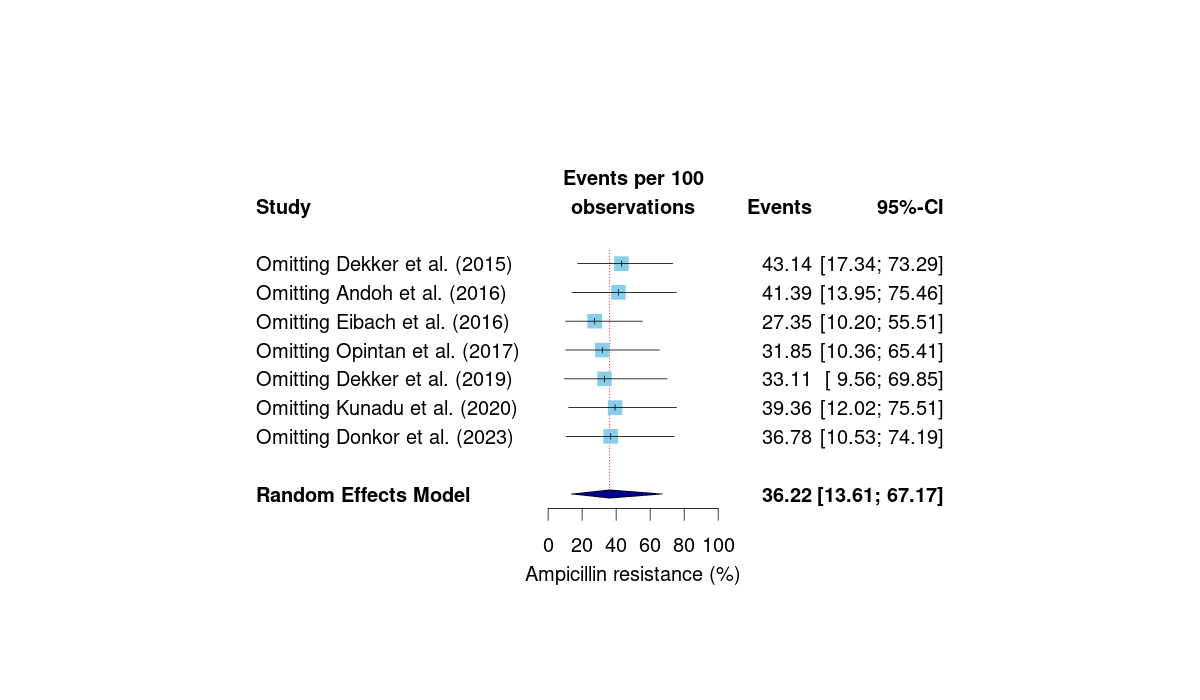
SF 20: Figure of influential analysis for ampicillin


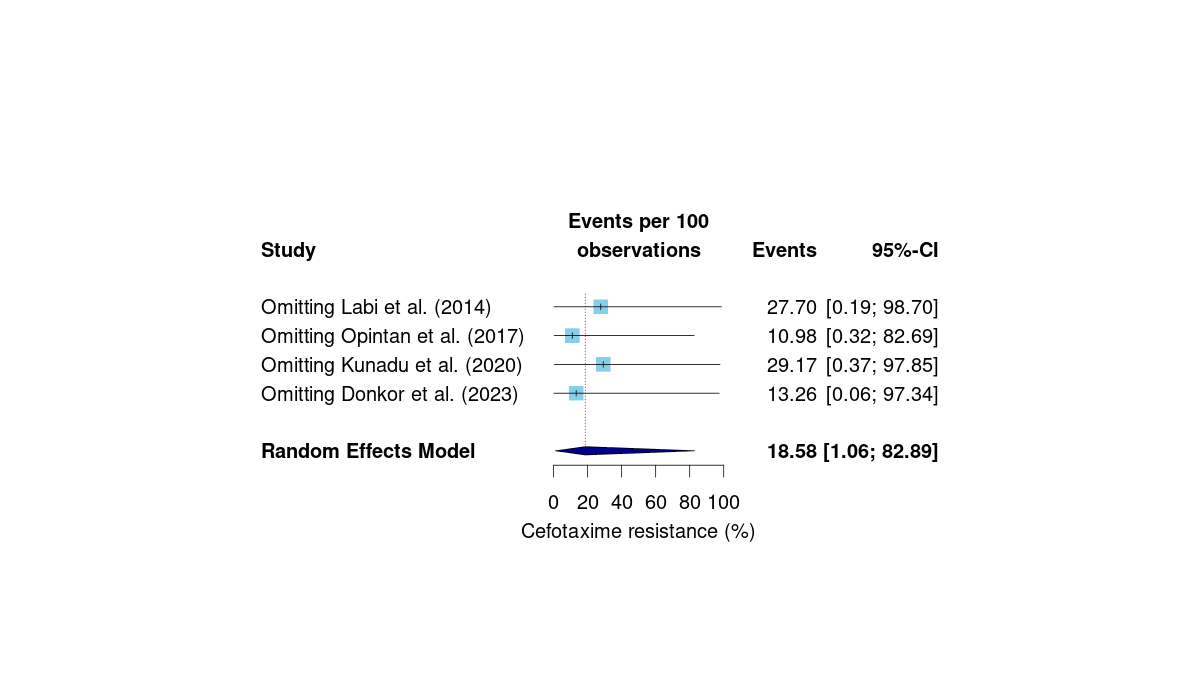
SF 21: Figure of influential analysis for cefotaxime


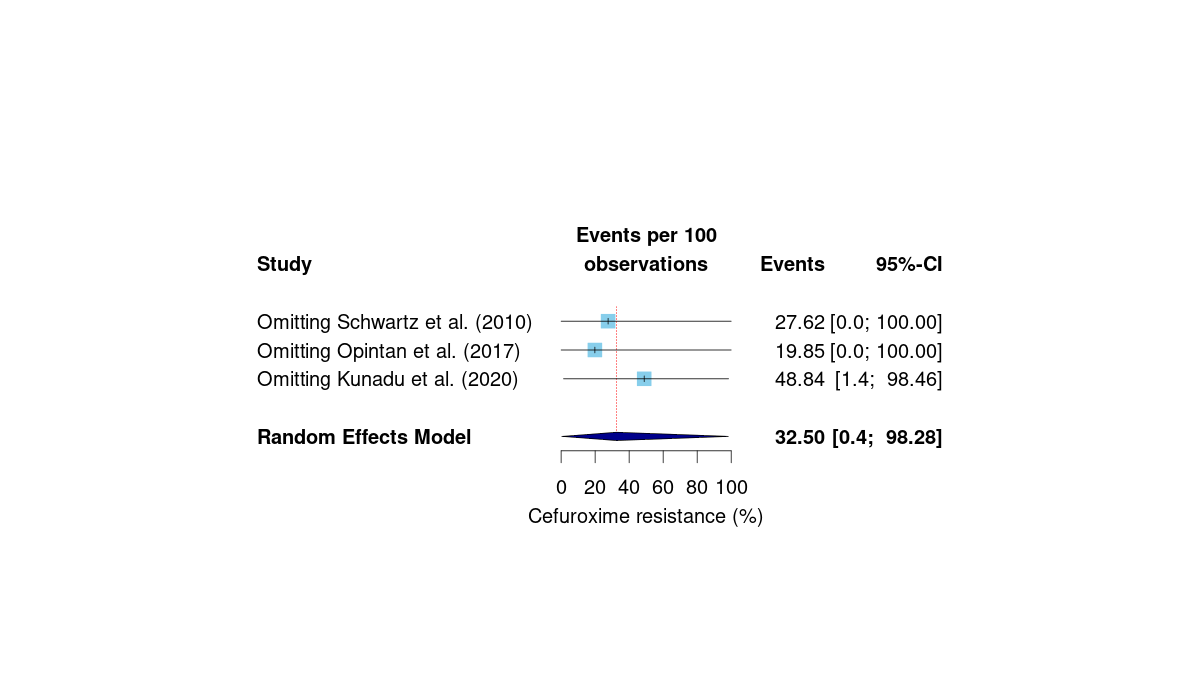
SF 22: Figure of influential analysis for cefuroxime


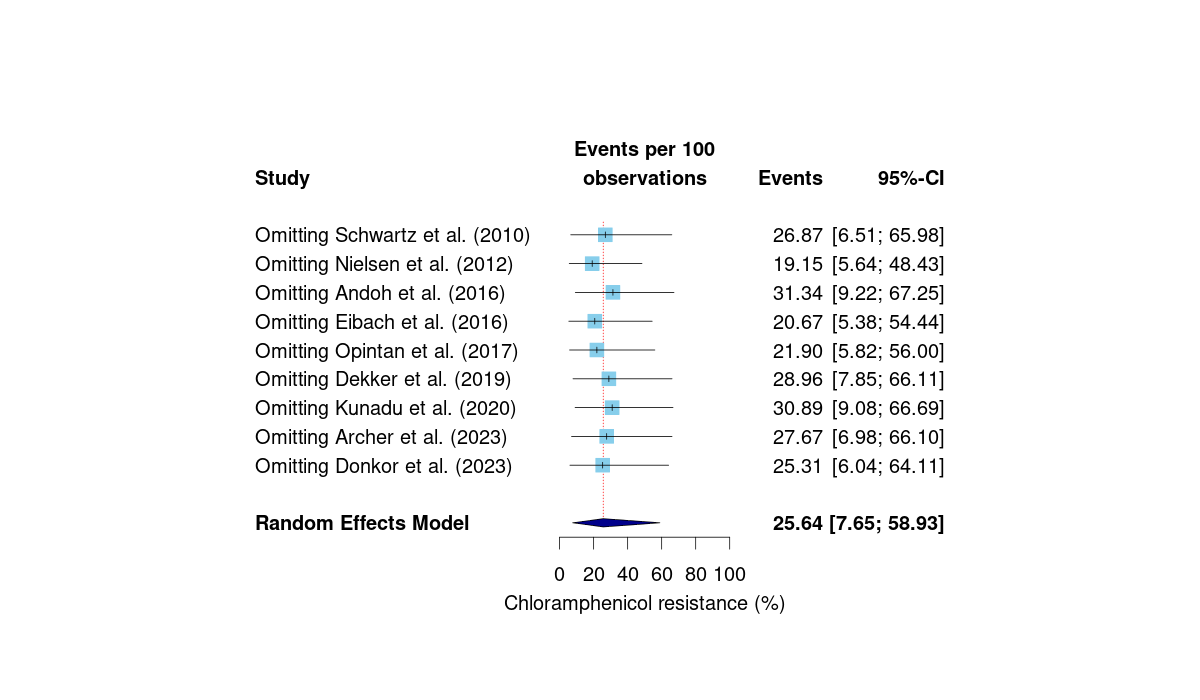
SF 23: Figure of influential analysis for chloramphenicol


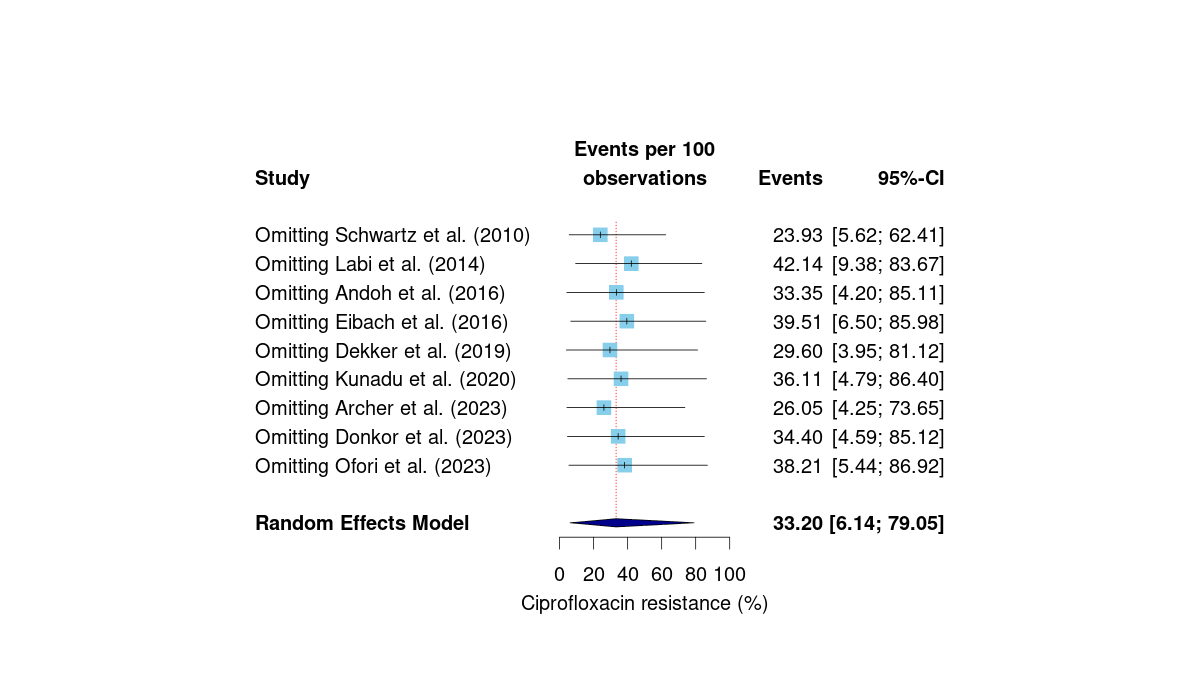
SF 24: Figure of influential analysis for ciprofloxacin


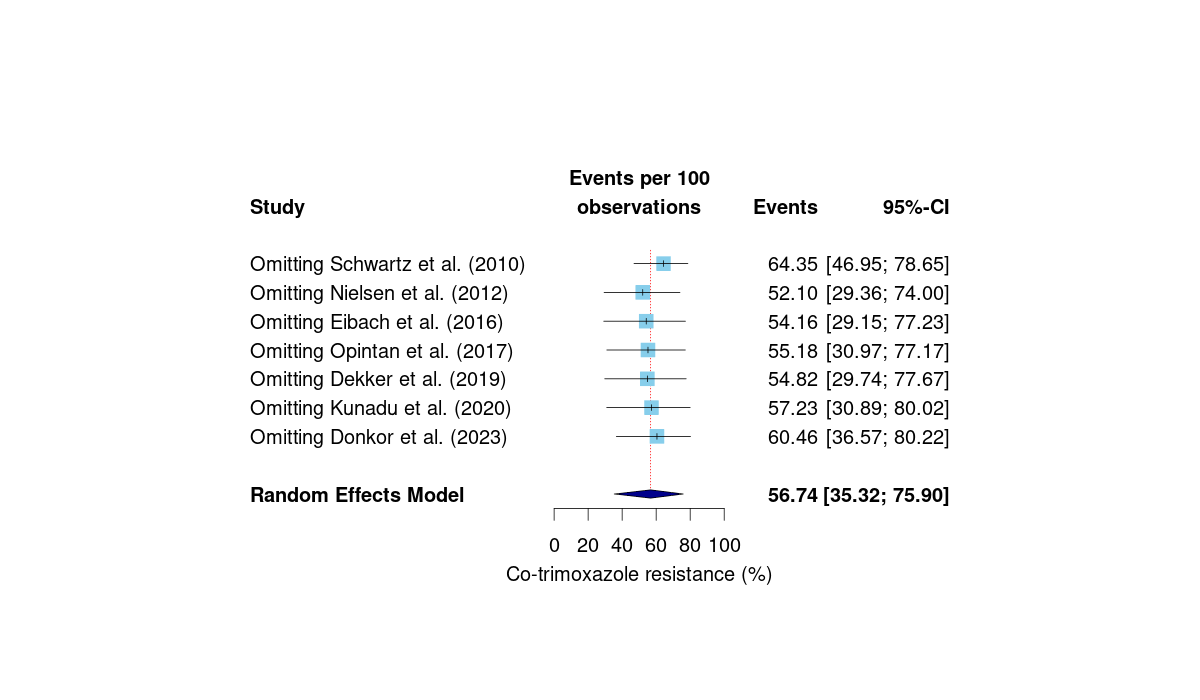
SF 25: Figure of influential analysis for co-trimoxazole


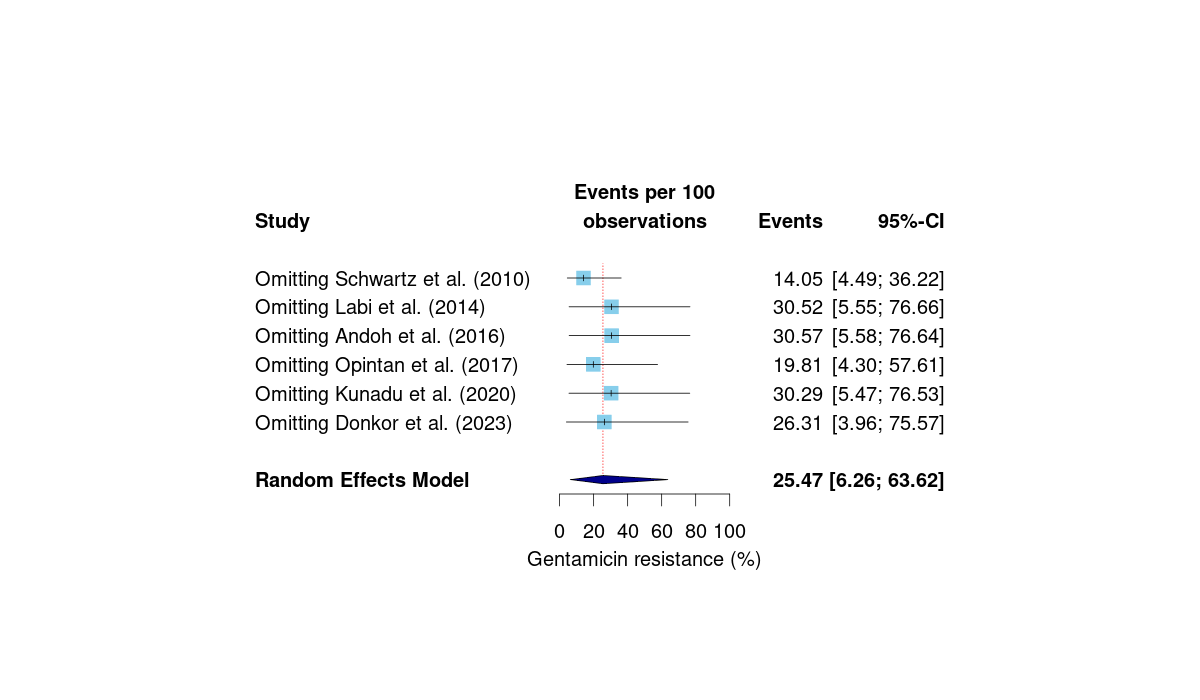
SF 26: Figure of influential analysis for gentamicin

SF 26: Figure of influential analysis for
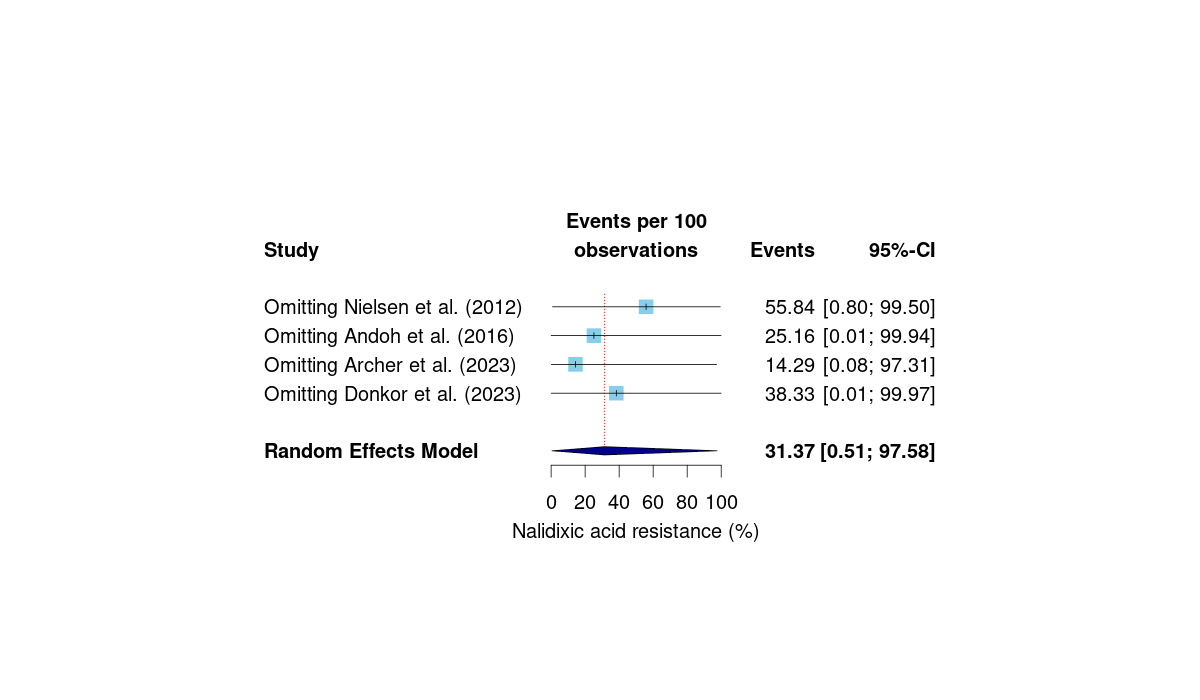
nalidixic acid


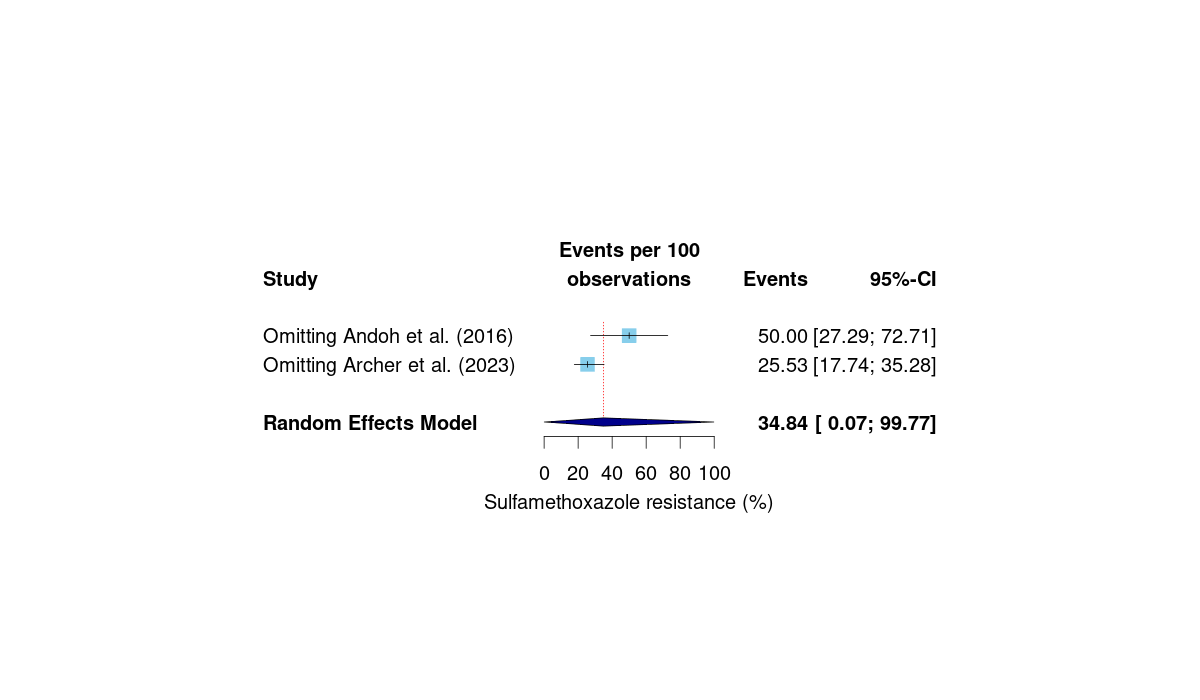
SF 27: Figure of influential analysis for sulfamethoxazole


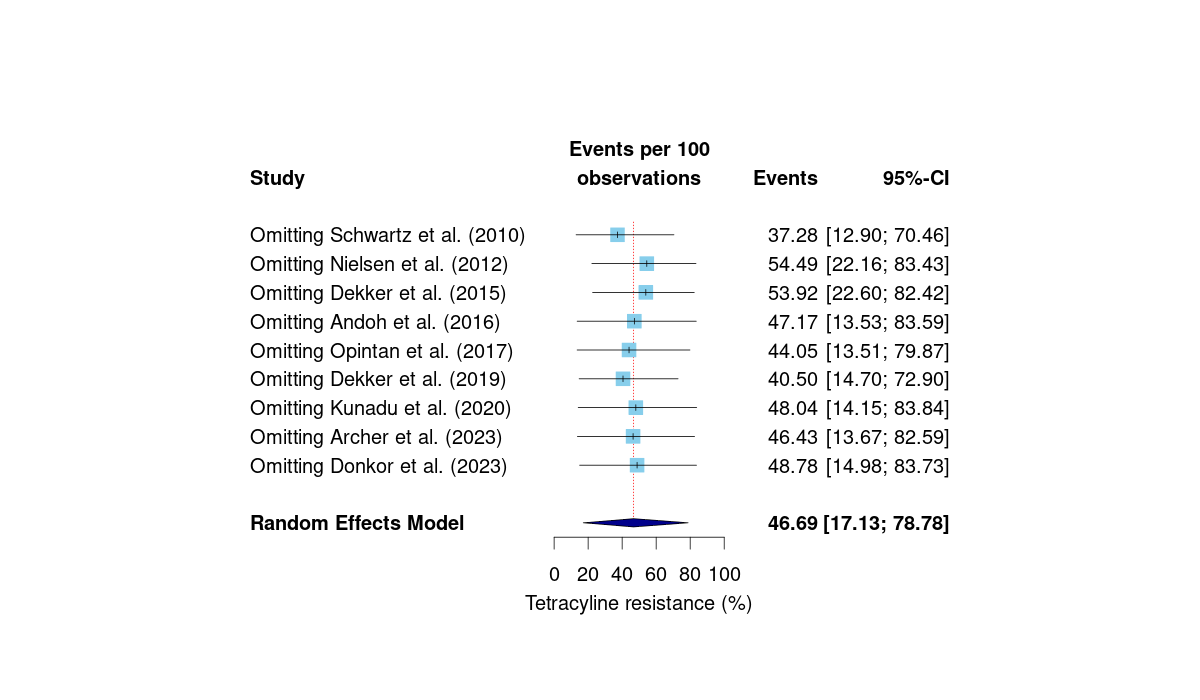


SF 28: Figure of influential analysis for tetracyline


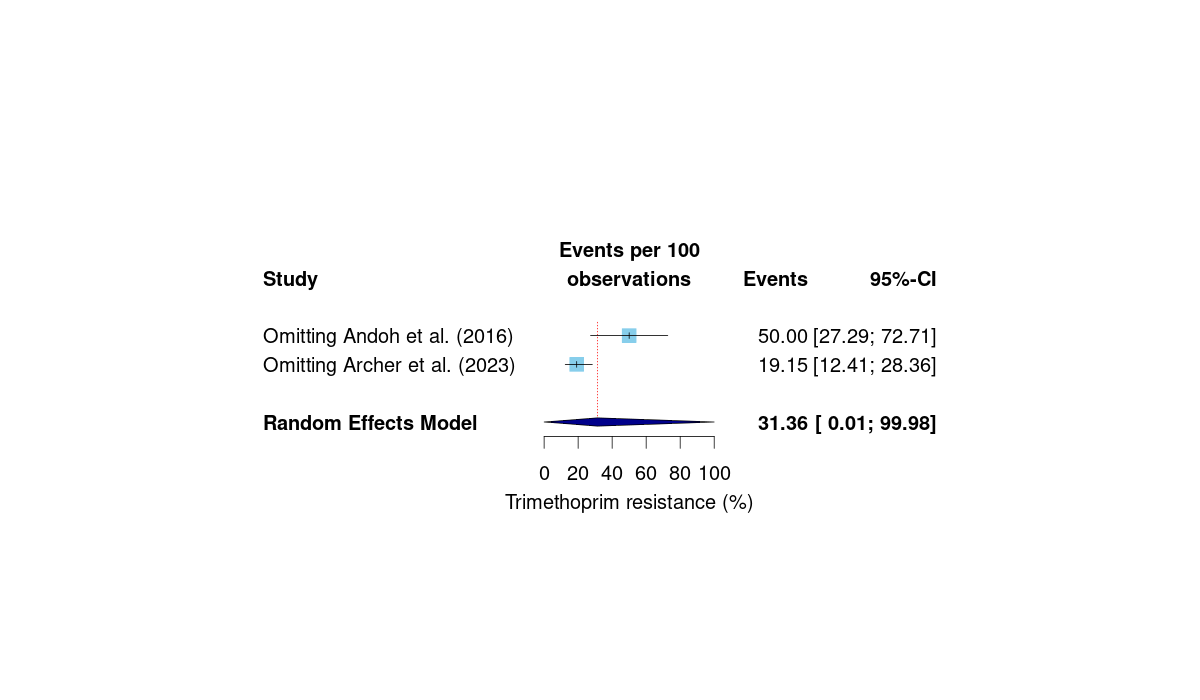
SF 29: Figure of influential analysis for trimethoprim
